# Long-term PGC1β overexpression leads to apoptosis, autophagy and muscle wasting

**DOI:** 10.1038/s41598-017-10238-9

**Published:** 2017-08-31

**Authors:** Danesh H. Sopariwala, Vikas Yadav, Pierre-Marie Badin, Neah Likhite, Megha Sheth, Sabina Lorca, Isabelle K. Vila, Eun Ran Kim, Qingchun Tong, Min Sup Song, George G. Rodney, Vihang A. Narkar

**Affiliations:** 1 Metabolic and Degenerative Diseases, Institute of Molecular Medicine, The University of Texas McGovern Medical School, Houston, TX 77030 USA; 2 Department of Biochemistry and Cell Biology, Rice University, Houston, TX 77005 USA; 3 Molecular and Cellular Oncology, The University of Texas MD Anderson Cancer Center, Houston, TX 77030 USA; 4 Graduate School of Biomedical Sciences at The University of Texas Health Science Center at Houston, Houston, TX 77030 USA; 5 Department of Molecular Physiology and Biophysics, Baylor College of Medicine, Houston, TX 77030 USA; 6 Integrative Biology and Pharmacology, The University of Texas McGovern Medical School, Houston, TX 77030 USA

## Abstract

Skeletal muscle wasting is prevalent in many chronic diseases, necessitating inquiries into molecular regulation of muscle mass. Nuclear receptor co-activator peroxisome proliferator-activated receptor co-activator 1 alpha (PGC1α) and its splice variant PGC1α4 increase skeletal muscle mass. However, the effect of the other PGC1 sub-type, PGC1β, on muscle size is unclear. In transgenic mice selectively over-expressing PGC1β in the skeletal muscle, we have found that PGC1β progressively decreases skeletal muscle mass predominantly associated with loss of type 2b fast-twitch myofibers. Paradoxically, PGC1β represses the ubiquitin-proteolysis degradation pathway genes resulting in ubiquitinated protein accumulation in muscle. However, PGC1β overexpression triggers up-regulation of apoptosis and autophagy genes, resulting in robust activation of these cell degenerative processes, and a concomitant increase in muscle protein oxidation. Concurrently, PGC1β up-regulates apoptosis and/or autophagy transcriptional factors such as E2f1, Atf3, Stat1, and Stat3, which may be facilitating myopathy. Therefore, PGC1β activation negatively affects muscle mass over time, particularly fast-twitch muscles, which should be taken into consideration along with its known aerobic effects in the skeletal muscle.

## Introduction

Aging and other chronic diseases including cancer, diabetes, obesity, and chronic obstructive pulmonary disease (COPD) lead to severe muscle wasting, affecting mobility and overall health^1–3^. With no effective intervention that prevents muscle wasting, there is a need for understanding the molecular pathways involved in muscle size regulation and for developing new pre-clinical models to understand muscle wasting. A widely studied mechanism of muscle wasting is the proteasomal degradation of proteins by activation of E3 ubiquitin ligases, such as tripartite motif containing 63 (TRIM63), F box only protein 32 (FBXO32), FBXO40, and neural precursor cell expressed developmentally down-regulated protein 4 (NEDD4), which are induced in muscle wasting associated with denervation and prolonged immobilization^4^. Apoptosis is also activated in myofibers by cellular stress, injury and impaired mitochondrial function leading to muscle loss^5, 6^. Altered autophagy flux, either impaired or heightened, has been shown to negatively impact muscle mass^7^. As much as ubiquitin-proteasome degradation, apoptosis, and autophagy are established cellular phenomenon associated with muscle homeostasis, the transcriptional regulators that orchestrate these pathways remain to be fully defined.

Peroxisome proliferator-activated receptor gamma coactivator (PGC) is a family of transcriptional co-regulators consisting of three members including PGC1α, PGC1β, and PGC1 related co-activator (PRC). PGC1α remains the most widely studied isoform, followed by PGC1β; however, little is known about PRC. PGC1α controls various aspects of skeletal muscle function and health. PGC1α is induced in the muscle by endurance exercise^8, 9^, and its overexpression increases mitochondrial biogenesis, oxidative type I slow-twitch myofiber switch, and angiogenesis in the skeletal muscles, resulting in increased fatigue resistance and running endurance^10, 11^. Overexpression of PGC1α also affords protection in a murine model of Duchenne muscular dystrophy (DMD)^12^. Loss of muscle PGC1α may be responsible for metabolic dys-homeostasis, insulin resistance and diabetes^13^. Interestingly, PGC1α is also protectively involved in maintenance of muscle mass in models of muscle wasting through the repression of forkhead box O (Foxo) 3a, TWEAK-NFkB interaction, and atrogenic genes^14–16^. PGC1α4, an isoform of PGC1α generated from alternative promoter usage and splicing of the primary transcript, causes skeletal muscle hypertrophy and increases strength; and can prevent cancer-associated muscle wasting^17^. PGC1β is responsible for encoding the features of type 2X oxidative/glycolytic myofiber type and the associated oxidative capacity and mitochondrial biogenesis^18^. In contrast to PGC1α, PGC1β activates an anti-angiogenesis gene program in the skeletal muscle, which impairs muscle recovery from ischemic injury^19^. Transient overexpression of PGC1β in muscle by electroporation protects against denervation induced muscle wasting by inhibiting ubiquitin-mediated proteolysis^20^. Along these lines, muscle PGC1β mRNA expression is suppressed in conditions such as sepsis and dexamethasone treatment, where ubiquitin-mediated proteolysis is reciprocally active^21^. While PGC1β is dynamically regulated in muscle wasting and seems to be protective, how PGC1β affects skeletal muscle size, particularly in absence of disease, and underlying transcriptional adaptations involved have not been fully elucidated.

In this study, we investigate the potential role of PGC1β in the regulation of skeletal muscle mass and the related gene network it activates. Using skeletal muscle-specific PGC1β transgenic (PGC1β-TG) mice, we demonstrate that PGC1β overexpression causes a progressive decrease in muscle mass. The loss of muscle mass is associated with oxidative stress and activation of the gene program leading to apoptosis and autophagy-mediated loss of myofibers. Mechanistically, PGC1β induces a battery of master-transcriptional factors including E2F transcription factor 1 (E2f1), transformation related protein 53 (Trp53), activating transcription factor 3 (Atf3), and signal transducer and activator of transcription (Stat) 1, 2 and 3, which are known to be associated with apoptosis and muscle wasting^22–25^.

## Results

### Skeletal muscle PGC1β overexpression reduces muscle mass and weight gain

**Figure 1:**
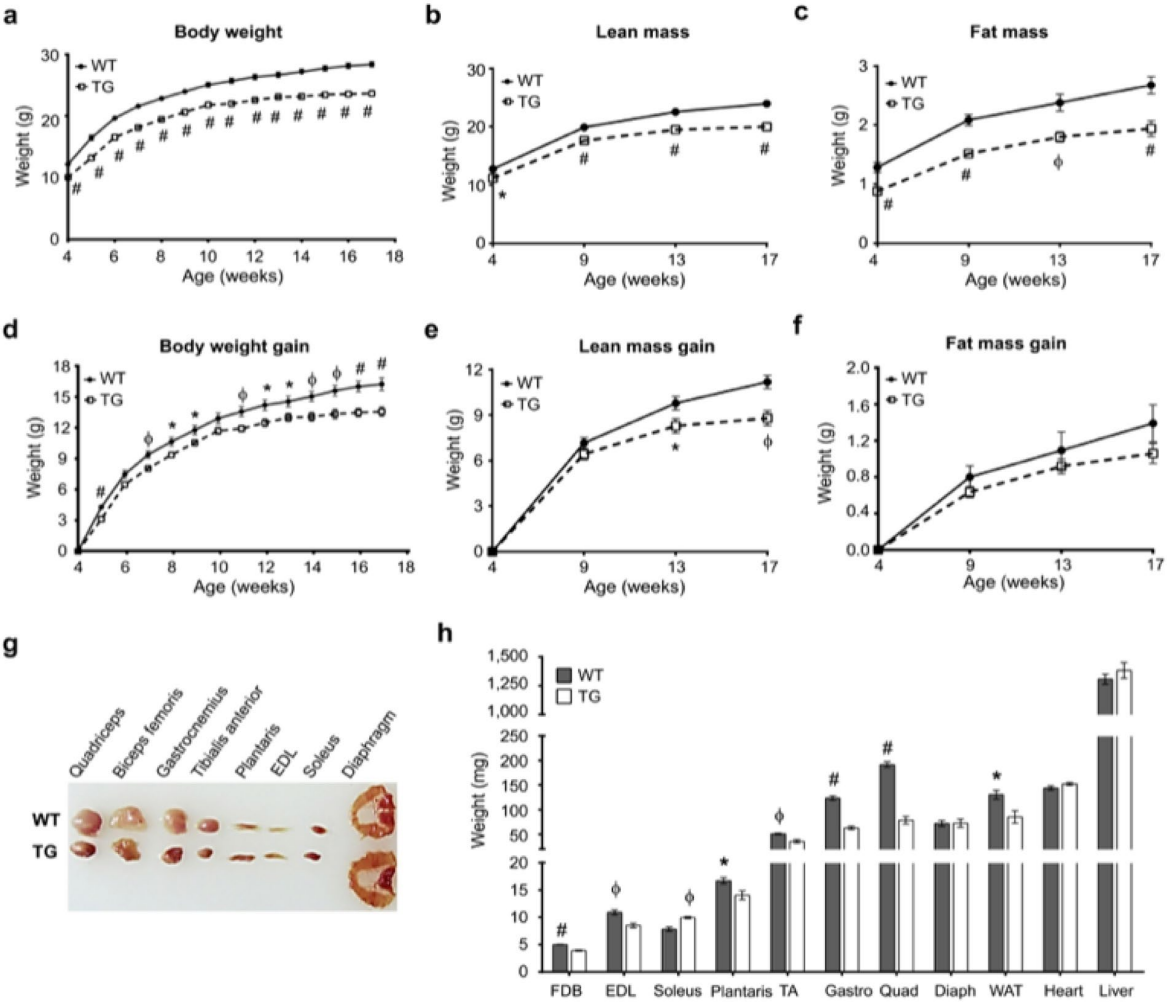
Body weight and muscle weights. Following parameters were measured in wild type (WT) and PGC1β-TG (TG) mice. **(a)** Whole body weight (n = 15). **(b)** Lean mass. **(c)** Fat mass. **(d)** Body weight gain (n = 15). **(e)** Lean mass gain (n = 15). **(f)** Fat mass gain (n = 15). **(g)** Representative images of various muscles. EDL – extensor digitorum longus. **(h)** Individual muscle weights of fast-twitch (FDB - Flexor digitorum brevis, EDL, TA – tibialis anterior, Quad – quadriceps, Gastro – gastrocnemius, and plantaris) and slow-twitch (soleus and Diaph – diaphragm) muscles, WAT - perigonadic white adipose tissue, heart and liver (n = 5 to 7). Data are represented as mean ± SEM. *p < 0.05, ^Φ^p < 0.01, and ^#^p < 0.001 (unpaired Student’s t test).

To investigate the role of PGC1β in the regulation of skeletal muscle mass, we used the muscle-specific PGC1β-TG mice, which we previously generated using the human skeletal alpha actin promoter^19^. The higher mRNA and protein expression of PGC1β in the muscles of PGC1β-TG mice compared to WT are shown in Supplementary Fig. S1. In PGC1β-TG compared to WT mice, the protein levels of PGC1β were ~5 fold higher in fast twitch muscles and 1.7 fold higher in the soleus, while expression in the heart was not affected. In comparing the PGC1β-TG and WT mice between 4–18 weeks of age, we found that the body weight, lean mass and fat mass were consistently lower in the transgenic compared to the wild type mice (Fig. 1a–c). When presented as ‘body weight gain’, the PGC1β-TG mice were found to gain significantly less weight compared to the WT mice (Fig. 1d). By 17 weeks of age, the PGC1β-TG mice weighed 16.75% less than WT mice [WT = 28.41 ± 0.44 g (n = 15), PGC1β-TG = 23.65 ± 0.50 g (n = 15), p < 0.0001]. The retarded weight gain in the PGC1β-TG mice correlated with a significantly lower gain in lean mass (Fig. 1e), and to a lesser extent a reduced gain in fat mass (Fig. 1f), since the latter is not significantly different between WT and PGC1β-TG mice, as determined by Echo MRI. Compared to WT mice, the muscle weights of the predominantly fast-twitch muscles [e.g. quadriceps, gastrocnemius, and tibialis anterior (TA)] from the PGC1β-TG mice were dramatically reduced, while there was no reduction in weights of the predominantly slow-twitch muscles (soleus and diaphragm) (Fig. 1g and h) from the same mice. We did not find a difference in the weights of heart and liver (Fig. 1h), where the transgene is not expressed. However, perigonadic white adipose tissue showed significant reduction in weight (Fig. 1h), consistent with the reduced fat mass of PGC1β-TG mice (Fig. 1c), likely due to PGC1β-mediated increase in oxidative metabolism in the skeletal muscle.

**Figure 2:**
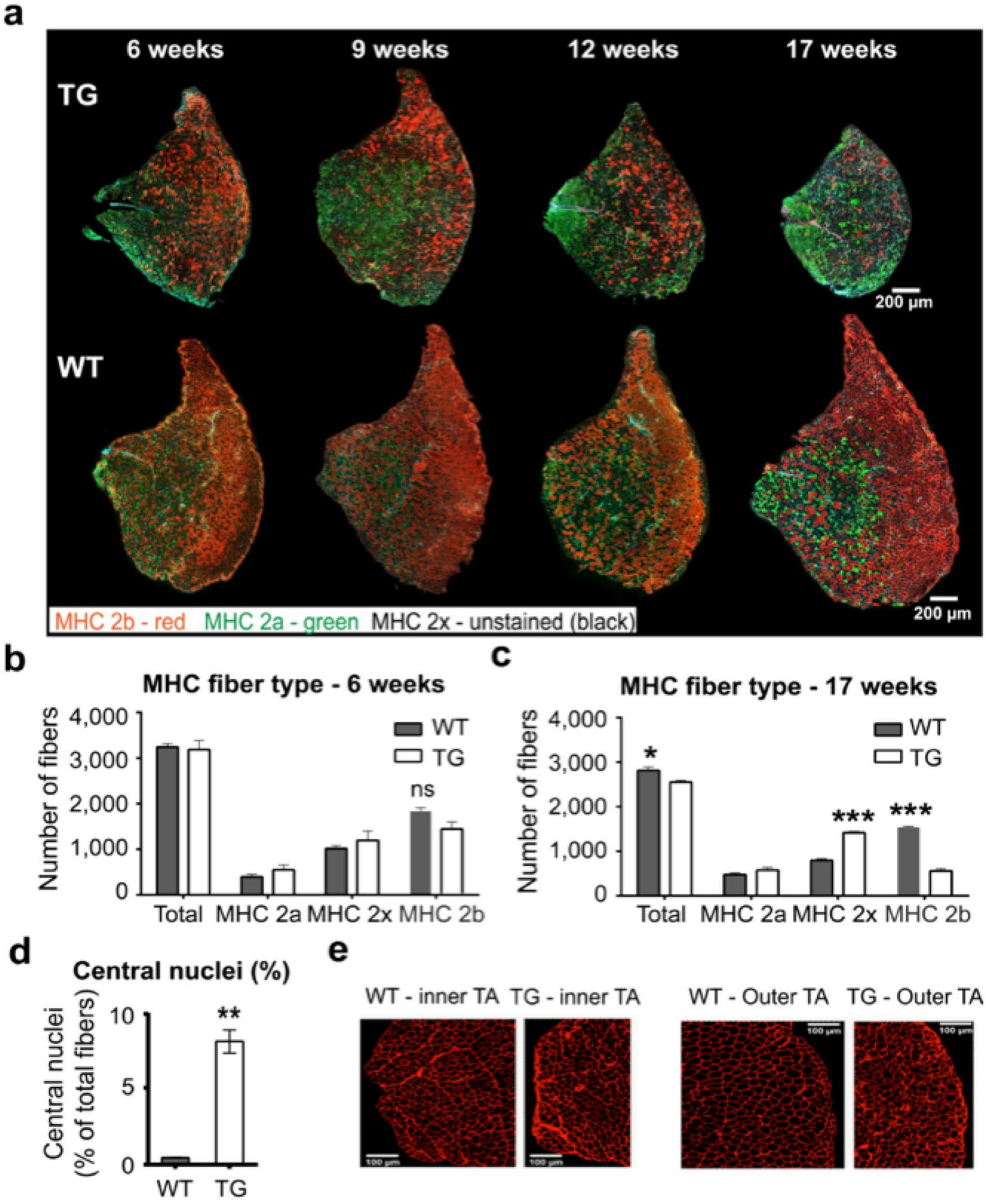
Myofiber typing in young and adult mice. **(a)** Representative images of myofiber type staining in TA from wild type (WT) and PGC1β-TG (TG) mice at different ages. Red – myosin heavy chain (MHC) 2b, Green – MHC 2a, and unstained myofibers – MHC 2x (n = 3 to 4). **(b,c)** Quantification of myofiber type distribution in TA of 6 week old (**b**) and 17 week old (**c**) mice. **(d)** Percent myofibers with central nuclei in 17 week old mice (n = 4) mice per group. **(e)** Representative images of laminin-stained cross-sections from inner and outer TA from 17 week old WT and TG mice. Data are represented as mean ± SEM. ns = not significant, *p < 0.05, **p < 0.01 and ***p < 0.001 (unpaired Student’s t test).

Because the loss in muscle mass was predominantly in fast-twitch muscles, we measured the effect of PGC1β overexpression on myofiber type distribution in TA muscles from mice between 6 to 17 weeks of age. PGC1β overexpression caused a progressive loss of fast-twitch myofibers, especially the myosin heavy chain (MHC) type 2b myofibers (Fig. 2a). In 6 week old mice, we did not see a significant difference in the total number of myofibers, as well as in the number of MHC 2a, 2x and 2b myofibers between PGC1β-TG and WT mice (Fig. 2b), although there was a non-significant trend for MHC 2b myofibers to be lower in PGC1β-TG TA (p = 0.064). By 17 weeks of age there was a 9.7% reduction in the total number of myofibers in the PGC1β-TG TA compared to the WT TA [WT = 2813 ± 75 (n = 4) and PGC1β-TG = 2552 ± 43 (n = 4), p = 0.0237] (Fig. 2c). The TA muscles from the 17 week old PGC1β-TG mice were composed of a significantly lesser number of MHC 2b myofibers [WT = 1539 ± 20 (n = 4) and PGC1β-TG = 559 ± 52 (n = 4), p < 0.0001] and significantly more MHC 2x myofibers [WT = 797 ± 40 (n = 4) and TG = 1414 ± 28 (n = 4), p < 0.0001] compared to the WT mice. The MHC 2a myofiber number was not significantly different between the two groups. The loss in myofibers does not seem to be because of an inability of PGC1β-TG muscles to form new myofibers, because the PGC1β-TG TA exhibited a 17.67 fold increase in percentage of central nuclei compared to the WT TA [WT = 0.46 ± 0.03% (n = 4) and TG = 8.17 ± 0.77% (n = 4), p = 0.0022] (Fig. 2d), indicative of recently regenerated myofibers.

**Figure 3:**
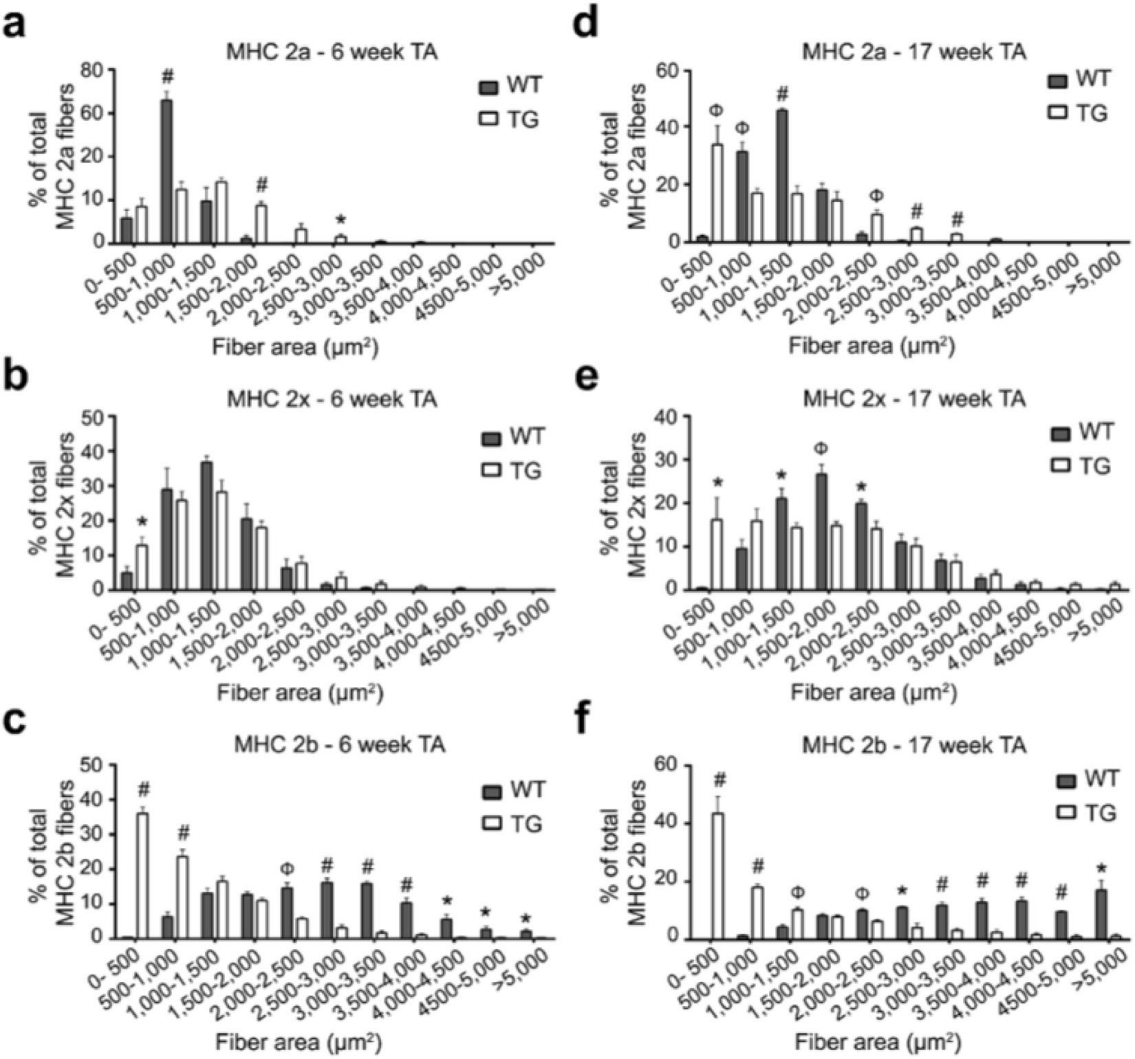
Myofiber area distribution of individual MHC myofibers in young and adult mice. **(a)** Quantification of MHC 2a myofibers as myofiber area vs. number distribution represented as a percentage of total MHC 2a myofibers in TA from 6 week old WT and TG mice. **(b)** Quantification of MHC 2x myofibers as myofiber size vs. number distribution. **(c)** Quantification of MHC2b myofibers as myofiber area vs. number distribution in 6 week old WT and TG TA. **(d–f)** Quantification of myofiber area vs. number distribution for MHC 2a (**d**), MHC 2x (**e**) and MHC 2b (**f**) myofibers in TA from 17 week old WT and TG mice. (n = 4 mice per group). Data are represented as mean ± SEM. *p < 0.05, ^Φ^p < 0.01, and ^#^p < 0.001 (unpaired Student’s t test).

We found that the myofiber loss is also accompanied by atrophy of the remaining myofibers as seen by smaller size of myofibers in the outer TA (predominantly MHC 2b myofiber) as well as inner TA (mostly MHC 2a and MHC 2x myofibers) of 17 week old PGC1β-TG compared to WT mice (Fig. 2e). To further characterize the muscle atrophy, we performed detailed analysis of myofiber area distribution of individual myofiber types in TA from young (6 week old) and adult (17 week old) mice. In 6 week old mice, we found that there was a leftward shift in the size of the MHC 2b myofiber in the PGC1β-TG compared to WT TA (Fig. 3c), indicative of atrophy. The MHC 2x myofibers also had a modest leftward shift in the 6 week old PGC1β-TG mice (Fig. 3b); however, the MHC 2a myofibers tends to show a rightward shift in the PGC1β-TG TA compared to WT TA (Fig. 3a). The rightward shift of MHC 2a myofiber area indicates that these slower myofibers are protected against atrophy and exhibit compensatory increase in size in the young PGC1β-TG TA. This is similar to the increase in overall mass seen in the PGC1β-TG soleus (a slow muscle composed of predominantly MHC 2a and MHC I myofibers) (Fig. 1h). Therefore, in the 6 week old mice, the smaller muscle size of PGC1β-TG TA, in spite of similar myofiber number as WT TA, is largely due to atrophy of MHC 2b myofibers in the PGC1β-TG compared to WT TA. By 17 weeks of age, there is significant atrophy in the PGC1β-TG TA as seen by the leftward shift in the MHC 2x (Fig. 3e) and MHC 2b (Fig. 3f) myofibers compared to WT TA. The MHC 2a myofibers from PGC1β-TG TA (Fig. 3d) show a mixed profile with some of them showing a significantly leftward shift (0–500 µm^2^) while others show a rightward shift (2,000–3,500 µm^2^) compared to WT. MHC 2b myofibers from PGC1β-TG TA are most severely affected and a majority of the surviving MHC 2b myofibers also show a significant leftward shift in size. Therefore, the progressive loss of muscle mass in PGC1β-TG mice is a summation of MHC 2b myofiber atrophy/loss and atrophy of other myofiber types, with a likely compensatory hypertrophy in MHC 2a myofibers. Furthermore, the atrophy of MHC 2b myofibers begins as early as 6 weeks of age in the transgenic mice.

### Incomplete ubiquitin-proteasomal degradation pathway in the PGC1β overexpressing muscles

To determine the potential cause of muscle wasting in the PGC1β-TG mice, we examined the gene microarray data we previously published from the TA of the WT and PGC1β-TG mice (NCBI GEO # GSE58699)^19^. The significantly regulated genes identified using the previously described criteria were subjected to KEGG pathway analysis using Genecodis^19, 26^. This analysis revealed three major pathways that are associated with muscle wasting, namely ubiquitin-mediated proteolysis (55 genes), apoptosis (31 genes) and autophagy (lysosome – 65 genes, phagosome – 66 genes). Since ubiquitin-mediated proteolysis plays a major role in a variety of conditions causing muscle wasting, we first examined the genes belonging to this pathway. A majority of the significantly regulated genes (39 out of 55) belonging to ubiquitin-mediated proteosomal degradation were downregulated in the PGC1β-TG compared to WT muscles (Supplementary Fig. S2a). Furthermore, mRNA expression of key genes of E3 ubiquitin ligase mediated proteolysis like *Fbxo32*, *Fbxo40, Trim63*, and *Nedd4* were significantly repressed in the TA and gastrocnemius muscles of the PGC1β-TG compared to WT mice (Supplementary Fig. S2b and c). We found that accumulation of ubiquitinated proteins was dramatically increased in the skeletal muscles of 6 and 22 week old PGC1β-TG compared to WT mice (Supplementary Fig. 2d). This change is likely indicative of an incomplete degradation program in transgenic muscle via ubiquitin-mediated proteolysis.

### Overexpression of PGC1β activates programmed cell death in myofibers

**Figure 4:**
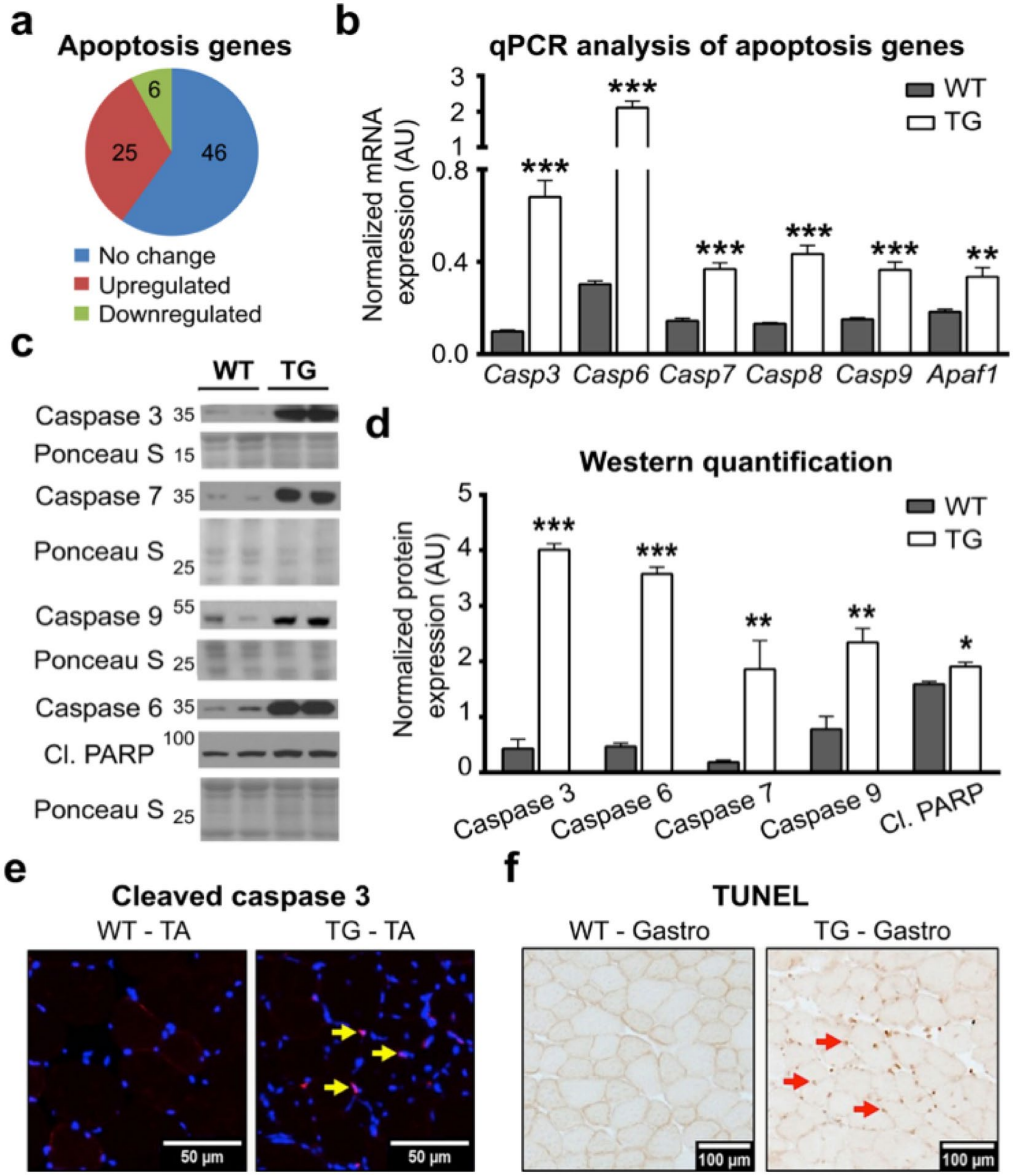
Measurement of apoptosis. Following were measured in TA muscles from 17 week old wild type (WT) and PGC1β-TG (TG) mice. **(a)** Pie chart representation of apoptotic gene regulation (n = 3). **(b)** mRNA expression of caspases and Apaf1 (n = 5). mRNA expression is normalized to *Eef2*. **(c)** Representative western blots of Caspase 3, 7, 9, 6 and cleaved PARP protein expression. **(d)** Quantification of western blots from (**c**) (n = 3 to 6). Protein expression is normalized to Ponceau S. **(e)** Representative cross-sectional image of TA showing immunofluorescence staining for cleaved caspase 3 (magenta) (n = 3). The staining is visible only in TG muscle sections; it co-localizes with the nucleus (blue) where it appears pink and is indicated with yellow arrows. **(f)** Representative image from TUNEL staining of gastrocnemius (Gastro) cryosections showing nicked (damaged) DNA in TG (brown spots indicated by red arrows) compared to WT muscles (n = 3). Data are represented as mean ± SEM. *p < 0.05*, **p < 0.01 and ***p < 0.001 (unpaired Student’s t test). Also see Supplementary Fig. S7.

Out of 31 cell death and apoptosis genes that were significantly regulated by PGC1β, 25 genes were induced (Fig. 4a). The up-regulated genes are listed in Supplementary Table S1. We further confirmed this finding by analyzing apoptosis related genes by qPCR and western immunoblotting. We found that there was an induction of caspases 3, 6, 7, 8, 9 and apoptotic protease activating factor 1 (Apaf1) in the TA (Fig. 4b) and gastrocnemius (Supplementary Fig. S3a) of PGC1β-TG mice. This data was also supported by protein expression analysis, where there was a significantly higher expression of caspases in the PGC1β-TG TA (Fig. 4c and d) and gastrocnemius (Supplementary Fig. S3b and c). To assess whether there was activation of apoptosis in the muscles, we quantified the protein expression of cleaved poly ADP ribose polymerase (PARP) and found that it was also significantly higher in PGC1β-TG muscles (Fig. 4c and d) (Supplementary Fig. S3b and c). PARP is involved in repairing damaged DNA and is one of the targets of cleaved caspase 3, the activated form of caspase 3^27^. We also looked at cleaved caspase 3 by immunofluorescence staining and found that there was co-localization of cleaved caspase 3 within the nucleus of the PGC1β-TG TA, but it was not detected in WT TA (Fig. 4e). Finally, we performed terminal deoxynucleotidyl transferase dUTP nick end labeling (TUNEL) staining on muscle sections and detected fragmented DNA in PGC1β-TG but not in WT gastrocnemius (Fig. 4f). Collectively, these data indicate that there is robust activation of the apoptotic cell death gene program in PGC1β-TG muscles, which may be responsible for the reduced number of myofibers seen in these muscles.

### Autophagy is elevated in PGC1β-TG muscles

**Figure 5:**
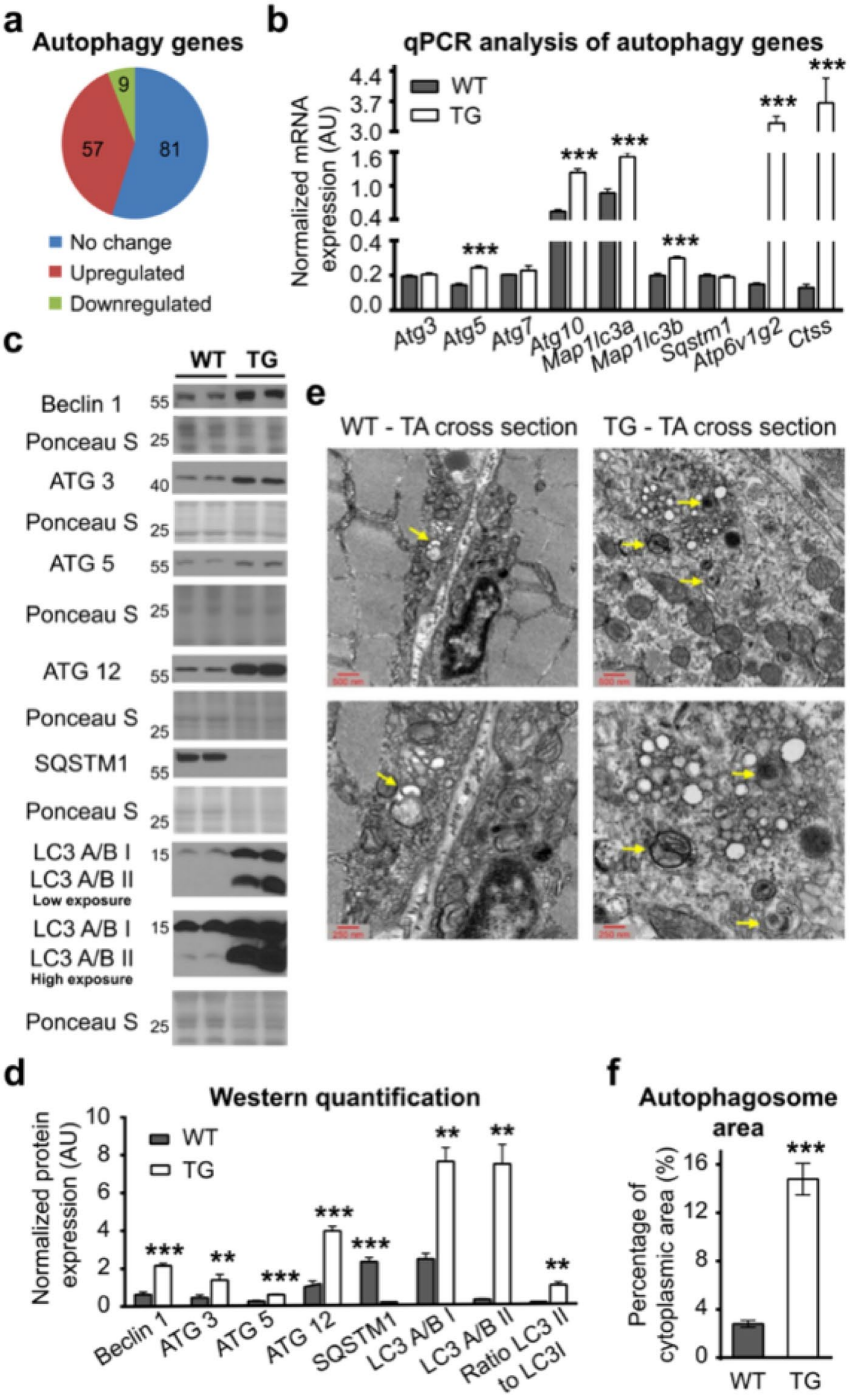
Measurement of autophagy. Following were measured in muscles from 17 week old wild type (WT) and PGC1β-TG (TG) mice. **(a)** Pie chart representation of autophagy gene regulation (n = 3). **(b)** mRNA expression of candidate autophagy and lysosomal genes in gastrocnemius (n = 5). mRNA expression is normalized to *Eef2*. **(c)** Representative western blots showing protein expression of Beclin1, Autophagy related (ATG) 3, 5, 12, LC3A/B I and II, and Sequestosome 1 (SQSTM1) in TA. **(d)** Quantification of protein expression in (**c**) and ratio of LC3 II to LC3 I (n = 3 to 6). Protein expression is normalized to Ponceau S. **(e)** Representative electron microscopy images of cross-sections of TA magnified at 25,000x (upper panel) and 50,000x (lower panel) showing double membrane autophagosomes with different cargo (yellow arrows). **(f)** The combined area of autophagosomal structures as a percentage of cytosolic area (n = 18 to 21 total micrographs at 25,000X magnifications per group from n = 3 TA per group). Data are represented as mean ± SEM. *p < 0.05, **P < 0.01, and ***p < 0.001 (unpaired Student’s t test). Also see Supplementary Fig. S7.

Out of the 66 genes that were significantly regulated by PGC1-β in the phagosome pathway, 57 were up-regulated (Fig. 5a and Supplementary Table S2). In addition, 53 of the 65 lysosomal genes regulated by PGC1-β were also up-regulated (Supplementary Table S3). Representative genes from each stage of the autophagy process, namely, phagophore formation (*Becn1)*, autophagosome elongation and formation (*Atg3, 5, 10, 12*, and *Map1lc3a and b*), and autophagolysosome degradation (*Atp6v1g2, Ctss*) were significantly induced at the mRNA and/or protein level in PGC1β-TG compared to the WT TA (Fig. 5b–d) and gastrocnemius (Supplementary Fig. S4) muscles. Microtubule associated protein 1 light chain 3 A and B (MAP1LC3A and B), henceforth referred to as LC3A and LC3B, belong to the autophagy related 8 (Atg8) conjugation system and are necessary for autophagosome formation^28^. LC3 needs to be conjugated to a phosphatidylethanolamine to be able to associate with the membrane; therefore, levels of the unconjugated (LC3 I) and conjugated (LC3 II) forms are often used as markers for autophagy flux^29^. Compared to WT, the PGC1β-TG muscles have higher expression of LC3A/B II to LC3A/B I (Fig. 5c and d) (Supplementary Fig. S4b and c), which is an indicator of higher autophagy flux. Sequestosome 1 (SQSTM1), which carries protein cargo to the forming autophagosome and is retained in the autophagolysosome, is eventually also degraded along with the other contents of the lysosome. Hence, monitoring the protein level of SQSTM1 serves as an indicator of completed autophagy^29^. PGC1β-TG TA (Fig. 5c and d) and gastrocnemius (Supplementary Fig. S4b and c) had significantly lower SQSTM1 protein than the respective WT muscles, while the mRNA levels were not lower (Fig. 5b and Supplementary Fig. S4a). Next, we used electron microscopy (EM) to visually observe the amount of autophagosomes and related structures present in the muscle. EM measurements showed that there was significantly more cytoplasmic area comprised of autophagosomes in the PGC1β-TG compared to the WT TA cross-sections (WT = 2.80 ± 0.28% (n = 18), TG = 14.78 ± 1.31% (n = 21), p < 0.0001) (Fig. 5e and f). These data taken together demonstrate that one of the mechanisms responsible for the higher muscle wasting in PGC1β-TG mice could be higher autophagy flux.

### PGC1β overexpression induces transcription factors upstream of apoptosis and autophagy pathways

**Figure 6:**
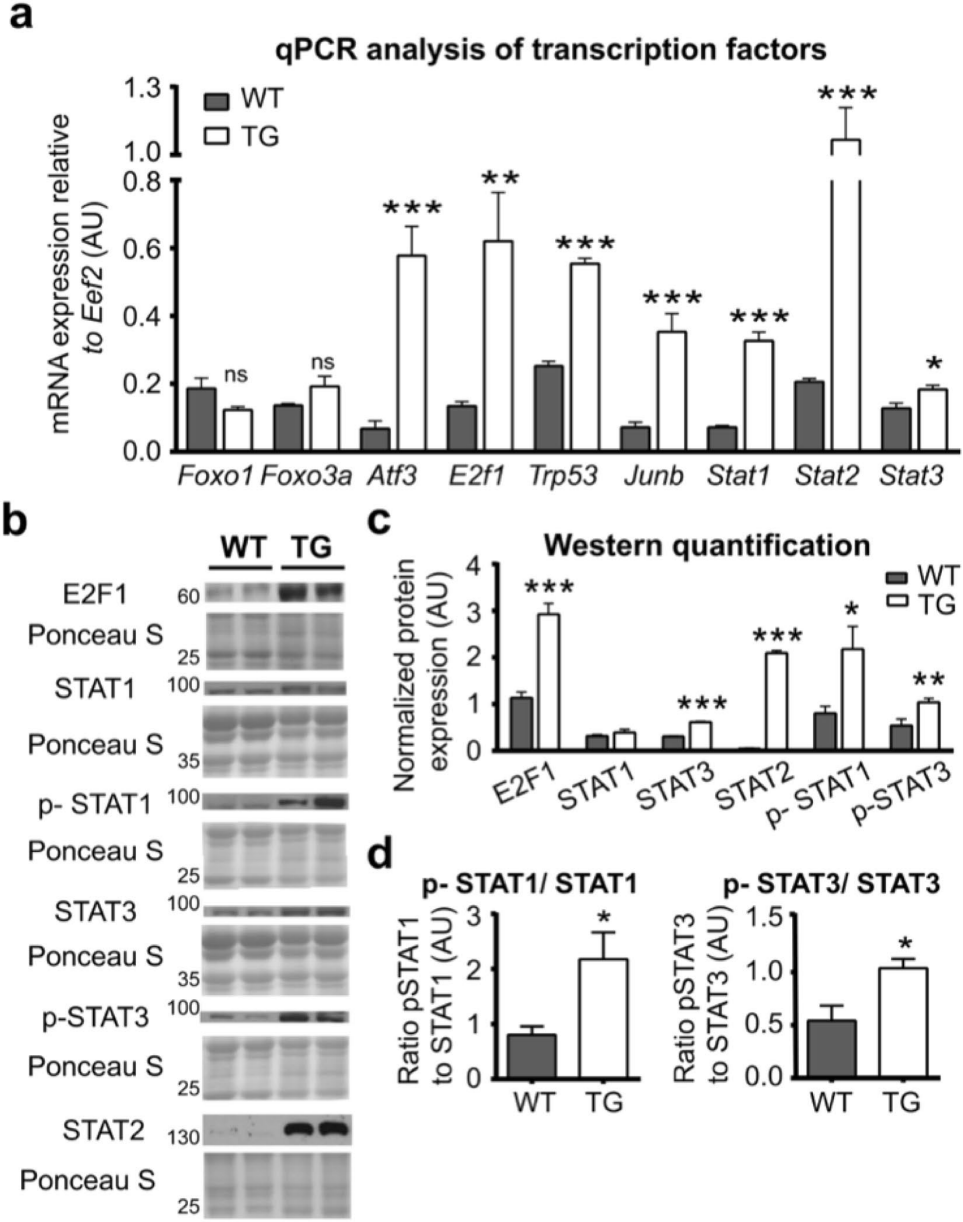
Expression of apoptosis and autophagy regulators. Following measurements were made in TA from wild type (WT) and PGC1β-TG (TG) mice. **(a)** mRNA expression of *Foxo1, Foxo3a, Atf3, E2f1, Trp53, Junb, Stat1, 2*, and *3*. **(b)** Representative western blot showing protein expression of E2F1, and phospho (p) and total STAT1, 3, and STAT2. **(c)** Quantification of western blots in (**b**). **(d)** Ratio of p-STAT1 to STAT1 and p-STAT3 to STAT3 (n = 4). Data are represented as mean ± SEM. ns = not significant, *p < 0.05, **p < 0.01, and ***p < 0.001 (unpaired Student’s t test). Also see Supplementary Fig. S7.

Because PGC1β activates a transcriptional program of apoptosis and autophagy, the co-activator may function through induction of master-transcriptional regulators of apoptosis and autophagy. We extended our screening of the gene array data from the WT and PGC1β-TG TA muscles to identify potential transcriptional factors that might be involved upstream to apoptosis and autophagy induction. We found that mRNA expression of several transcriptional factors involved in apoptosis (*E2f1, Atf3* and *Trp53*) was robustly induced in the TA muscles of PGC1β-TG mice (Fig. 6a)^23, 30^. Likewise, there was induction in the mRNA expression of *Stat 1, 2*, and *3* (Fig. 6a), which are also involved in muscle wasting^25, 31^. The protein expression of these transcriptional factors [e.g. E2F1, phospho-STAT1 Tyr701 (active form), STAT2, STAT3 and phospho-STAT3 Tyr705 (active form)] was also induced in the PGC1β-TG compared to the WT TA (Fig. 6b and c). Surprisingly, mRNA levels of *Foxo1* and *3a*, which are major regulators of apoptosis and autophagy, were not different between WT and PGC1β-TG muscles (Fig. 6a). The Akt/mTOR pathway, another key regulator of muscle mass and autophagy, was not repressed by PGC1β. We measured protein expression of Akt, phospho-AKT (Ser473), tuberous sclerosis (TSC) 2, phospho-TSC 2 (Thr1462), mammalian target of rapamycin (mTOR), phospho-mTOR (Ser2448) and downstream targets of mTOR (P70S6K and phospho-P70S6K Ser371), and found that they were either unchanged or higher in PGC1β-TG muscle as compared to WT (Supplementary Fig. S6).

### Progressive muscle protein oxidation in PGC1β-TG mice

**Figure 7:**
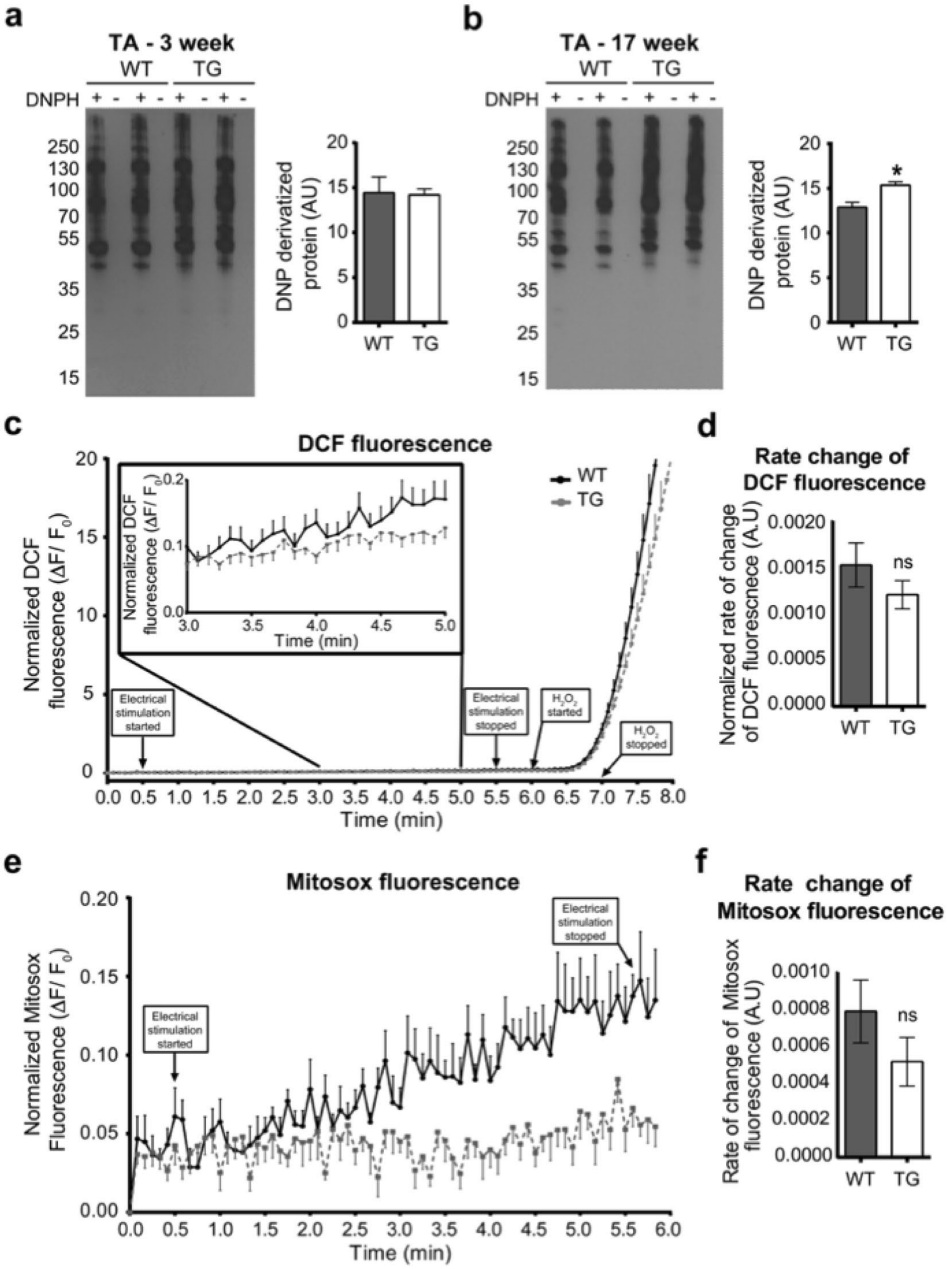
Measurement of oxidized proteins and reactive oxygen species (ROS) production. **(a,b)** Western blot and quantification showing levels of DNP (2,4 dinitophenyl hydrazone) derivatized oxidized proteins in 3 week old (**a**) and 17 week old (**b**) WT and PGC1β-TG tibialis anterior (TA). The “+” and “−” lanes indicate samples that have and that have not been derivatized with DNPH, respectively. (n = 3 to 4 mice per group). **(c)** Carboxydichlorofluorescein (DCF) fluorescence in flexor digitorum brevis (FDB) myofibers from 17 week old WT and TG mice in response to electrical stimulation and maximal H_2_O_2_ induction. Inset: Magnified view from time 3 min to 5 min, which was used to calculate slope. The arrows with boxed text indicate the time points at which stimulation was started and stopped, and when H_2_O_2_ perfusion was started and stopped (ΔF = F_t_ − F_0_ and F_0_ = Initial fluorescence) (n = 24 traces per group from 5 mice per group). **(c)** Quantification of rate of change of DCF fluorescence in response to stimulation (normalized to maximal rate of change in response to H_2_O_2_) measuring cytosolic ROS production between WT and TG FDB myofibers. **(e)** Graphical representation of change in Mitosox fluorescence in FDB myofibers from WT and TG mice representing mitochondrial ROS production in WT and TG muscles (n = 12 traces for WT and n = 13 traces for TG from 5 mice per group). **(f)** Rate of change of Mitosox fluorescence in WT and TG FDB myofibers. Data are represented as mean ± SEM. ns = not significant (unpaired Student’s t test).

PGC1β increases mitochondrial biogenesis as well as oxidative phosphorylation in the skeletal muscles. We previously showed that PGC1β-TG mice have higher succinate dehydrogenase muscle activity (indicative of oxidative capacity) compared to the WT mice^19^. The higher mitochondrial content in PGC1β-TG muscles correlates with the higher basal metabolic rates in these mice, as demonstrated by the increased oxygen consumption in PGC1β-TG mice (Supplementary Fig. S5a and b). Food consumption and ambulatory activity were not different between the two groups (Supplementary Fig. S5c and d). Increased fatty acid oxidation can cause oxidative stress leading to muscle atrophy^22^. Thus, we tested whether higher oxidative stress in PGC1β-TG muscles could be the cause of the observed apoptosis and autophagy by measuring protein oxidation by quantifying DNP derivatized oxidized proteins by western blot, as well as by measuring the rate of reactive oxygen species (ROS) production. Analysis of protein oxidation levels shows increased skeletal muscle protein oxidation in 17 week old PGC1β-TG TA but not in 3 week old PGC1β-TG TA as compared to age-matched WT mice (Fig. 7a and b) indicating that protein oxidation increases as atrophy and muscle loss get worse in the PGC1β-TG muscle. We also measured ROS production in isolated myofibers from flexor digitorum brevis (FDB) muscle. We used FDB myofibers for this study since it is a small muscle and thus is easier to work with and gives consistent results. Moreover, FDB myofibers are less prone to contraction-induced damage and hence are ideal for studying muscle function in the context of atrophy/damage. First, we confirmed that FDB muscle, similar to TA and gastrocnemius, undergoes atrophy in the PGC1β-TG compared to WT mice (Fig. 1h), and has significantly lesser MHC 2b myofibers (data not shown). We found that cytosolic ROS production, measured by monitoring carboxy-dichlorofluorescein (DCF) fluorescence, was comparable between PGC1β-TG and WT flexor digitorum brevis (FDB) myofibers during muscle contraction [WT = 0.001533 ± 0.0002383 (n = 24), TG = 0.001211 ± 0.0001538 (n = 24), p = 0.263] (Fig. 7c and d). Mitosox fluorescence was used to measure mitochondrial ROS production in response to electrical stimulation, and that was also similar between WT and PGC1β-TG myofibers [WT = 0.00079 ± 0.00017 (n = 12), TG = 0.00052 ± 0.00013 (n = 13), p = 0.215] (Fig. 7e and f). Lastly, we also looked at the different states of mitochondrial respiration in isolated mitochondria from WT and PGC1β-TG fast-twitch hind limb muscles to rule out impaired mitochondrial respiration and mitochondria-based oxidative stress as possible causes for muscle wasting. We found that there was no difference in State 3 (ADP-dependent), State 4 (in the presence of Oligomycin), and maximal respiration [in presence of Trifluoromethoxy carbonylcyanide phenylhydrazone (FCCP)] (Supplementary Fig. S5e) between mitochondria from the WT and PGC1β-TG muscles, indicating that mitochondrial quality was not altered by PGC1β overexpression. These data suggest that while there was an increase in muscle protein oxidation (indicative of oxidative stress) in older (17 week) PGC1β-TG mice compared to WT mice, there was no measurable increase in ROS production or, for that matter, aberrant mitochondria in PGC1β-TG mice. Nevertheless, increased oxidative stress might contribute to muscle wasting in PGC1β-TG mice, especially as atrophy progresses with age, and may act as a deterrent in the muscle’s ability to maintain its mass.

## Discussion

In this study, we have shown that sustained PGC1β overexpression in the skeletal muscle leads to progressive loss of muscle mass. PGC1β overexpression actually suppresses the ubiquitin-proteosomal degradation pathway, typically involved in muscle atrophy under various dilapidating conditions. Rather, long-term PGC1β activation causes transcriptional activation of apoptosis, autophagy, and subsequently oxidative stress, potentially via induction of key transcriptional factors involved in these two catabolic cellular phenomena.

While the role of PGC1α in the maintenance of muscle mass has been extensively explored, the precise role of PGC1β in regulating muscle size and the underlying transcriptional program had so far not been fully elucidated. PGC1α blocks immobilization as well as unloading induced autophagy and muscle wasting^32, 33^. Mitigation of aging-induced sarcopenia by PGC1α is linked with the repression of both apoptosis and autophagy in the skeletal muscle^34^. PGC1α however, has been reported to simulate autophagy in the skeletal muscle, particularly in the context of exercise^35, 36^. Long-term muscle-specific overexpression of PGC1α induced muscle atrophy due to mitochondrial uncoupling and ATP depletion^37^. Recently, Brault *et al*., showed that transient overexpression of PGC1β in muscle by electroporation protects against denervation induced muscle wasting by inhibiting ubiquitin-mediated proteolysis^20^. In another study, PGC1β mRNA expression in mice and rats was found to be decreased in response to sepsis and dexamethasone, where both conditions induce ubiquitin mediated proteolysis through up-regulation of Fbxo32 and Trim63 expression^21^. In agreement with these studies, we observed that the mRNA expression of E3 ubiquitin ligases (*Fbxo32* and *Trim63*), and expression of many genes in the ubiquitin-proteosomal degradation pathway are repressed by PGC1β, and might very well be responsible for the protective effects of transiently activated PGC1β. Our work shows that in contrast to transient activation, long-term overexpression of PGC1β leads to a progressive decrease in skeletal muscle mass. This effect coincides with the build-up of ubiquitinated proteins, activation of oxidative stress, apoptosis and autophagy, which altogether could be due to early and sustainable PGC1β activation in the transgenic skeletal muscle. Indeed, the transgene is even expressed in nascent myofiber, where the alpha-skeletal actin promoter is known to be active. It remains to be seen whether oxidative stress, and apoptosis or autophagy are not induced upon transient PGC1β overexpression resulting in an alternative protective effect.

It is noteworthy that in the young mice (6 week old), PGC1β overexpression does not affect myofiber type distribution, although there is detectable muscle atrophy in MHC 2b myofibers. It is only in the adult mice (17 week old) that we see significantly more MHC 2x and significantly less MHC 2b myofibers in PGC1β-TG compared to WT muscle, which is in agreement with previous studies^18, 19^. This apparent myofiber type switch is associated with a previously unappreciated reduction in total myofiber number arising from loss and atrophy of MHC 2b myofibers, along with atrophy of the MHC 2x myofibers in the PGC1β-TG muscle. Apoptosis and autophagy are the likely processes involved in myofiber loss.

Muscle cell death by apoptosis is a major contributor to muscle pathology associated with aging^38, 39^, muscular dystrophy^40, 41^, cancer cachexia^42^, and COPD^43^. Apoptosis is a complex yet coordinated process which results in programmed cell death and involves dissolution of the damaged nucleus^44^. The multinucleated composition of skeletal muscle myofibers means that myonuclear dissolution does not have to result in myofiber death. Apoptosis induction can be limited to clearance of damaged myonuclei along with the surrounding sarcoplasm, resulting in muscle atrophy. Indeed, in some instances apoptosis precedes muscle atrophy through ubiquitin-mediated proteolysis^45^. However, PGC1β-TG muscles exhibit a reduction in myofiber number, suggesting that these muscles are not subjected to partial apoptosis because there is death of the entire myofiber. The classical apoptosis pathway involves activation of caspase 8, which is required for apoptosome formation (Cytochrome C, Apaf1 and procaspase 9). This subsequently activates caspases 3, 6 and 7, which cleave the terminal targets of apoptosis, including PARP, resulting in DNA fragmentation and eventually leading to cell death^27^. Alternatively, caspase 8 can also directly activate caspase 3 forming cleaved caspase 3^27^. The PGC1β-TG muscles show up-regulation of major apoptosis genes leading to myofiber death. It is not clear what causes the initiation of apoptosis in the PGC1β-TG muscle, however studies have shown that even in the absence of the Fas ligand, caspase 8 can be recruited to Fas by Fas-associated protein with death domain (Fadd)^46, 47^, which is also induced in the PGC1β-TG TA (Supplementary Table S1).

Autophagy is an important catabolic process required for muscle homeostasis and plays a ‘pathway and pathological context’-dependent role in skeletal muscle health. For example, autophagy is necessary for preventing senescence and depletion of satellite cells^48^. Impaired autophagy can cause degeneration of the neuromuscular junctions and aging like symptoms in the muscle^49^. Autophagy can protect against muscle atrophy in type II glycogen storage disease^50^. Exercise can stimulate autophagy and enhance insulin sensitivity^51^. Activation of autophagy is also necessary for exercise-induced improvement in muscle function and performance^52^. On the other hand, autophagy can also be detrimental to muscle function. Autophagy-driven degradation is responsible for muscle wasting in cancer cachexia^47, 53^. FoxO3 can stimulate autophagy and cause muscle atrophy^54, 55^. Activation of signaling pathways such as AMP activated protein kinase (AMPK), Fyn/STAT3/Vps34, purinergic receptor 7 (P2RX7), TP53INP2, and Toll-like receptor 4 stimulates autophagy and induces muscle wasting^56–60^. Therefore, several signaling pathways converge on autophagy and its fine-tuning, including now PGC1β. A majority of the key mediators of the autophagy pathway (Becn1, Atg3, 5, 12, and Map1lc3b) are induced by PGC1β, suggesting that there is wide-scale activation of autophagy. However, the Akt/mTOR pathway, which is a major repressor of autophagy^61^, was intact in the PGC1β muscle, indicating that PGC1β does not function by targeting this pathway.

PGC1β up-regulates a battery of transcription factors known to control apoptosis and autophagy. E2F1 induces apoptosis through p53 dependent and independent pathways, simultaneously inhibiting anti-apoptotic signals^23, 62^. While E2F1 and p53 are up-regulated in PGC1β-TG muscle, there is also a down-regulation of E2F6 (E2F1 silencer) and mouse double minute 2 (p53 repressor) (data not shown)^63, 64^. The stress response transcription factor Atf3 is also induced in PGC1β-TG muscle and has been shown to co-localize and stabilize p53 while also activating it by blocking its ubiquitination^30, 65^. The p53-independent apoptosis is through transactivation of Apaf1 and a subsequent induction of caspases 3, 6, 7, and 9, all of which are up-regulated by PGC1β^23^. E2F1 was also shown to regulate autophagy and transcription of autophagy genes^24^. Junb, another transcription factor that is up-regulated by PGC1β (Fig. 6a), blocks activation of Fbxo32 and Trim63 by inhibiting FoxO3a, but does not have any effect on the autophagy-lysosome system^66^. FoxO1 and FoxO3a have been shown to play a crucial role in muscle mass regulation through transcription of E3 ubiquitin ligases and autophagy genes^7^; however, the gene expression of these factors was not affected by PGC1β. In recent studies STAT3 and, phospho-STAT3, which are induced by PGC1β, were shown to cause muscle atrophy in different types of cancer cachexia^31, 67^.

Because PGC1β simultaneously induced apoptosis and autophagy genes in the skeletal muscle, we reasoned that PGC1β might be targeting a common activator of these two cell degradation processes. We and others have previously reported that PGC1β induces mitochondrial biogenesis and oxidative phosphorylation^18, 19^ in the skeletal muscle^17, 18^. Mitochondria are sites of ROS production and elevated ROS levels have been associated with both apoptosis and autophagy^68–70^. However, PGC1β overexpression did not induce any detectable change in cytosolic and mitochondrial ROS production in muscle. In fact, there is a trend for reduced ROS production in our studies, which is in agreement with a recent study with PGC1β knockout mice showing increased ROS production in muscle^71^. Isolated mitochondrial respiratory parameters were also comparable between the WT and PGC1β-TG muscle, eliminating the likelihood of defective mitochondria as the causative factor. Nevertheless, we detected an increase in protein oxidation, suggestive of oxidative stress in the transgenic skeletal muscle of older 17 week old mice, but not in the younger 3 week old mice. The reason for the disconnection between ROS production and protein oxidation in our model is unclear. One likely possibility is that, the protein oxidation seen in older PGC1β-TG TA may be a consequence of the underlying apoptosis/atrophy, which would also explain why it is not observed in the young PGC1β-TG muscle.

In summary, we have shown that sustained skeletal muscle overexpression of PGC1β results in progressive loss of muscle mass, associated with both a decrease in muscle cross-sectional area and myofiber number. Transcriptionally, PGC1β activates a wide-scale gene program predominantly constituting activation of pro-apoptosis and autophagy genes, histo-morphologically resulting in increased apoptosis and autophagy in the skeletal muscle. The transcriptional factors or nuclear receptors mediating the PGC1β phenomenon remain to be identified. Our previous studies have shown that the anti-angiogenesis gene program activated by PGC1β involved activation of nuclear receptor COUP-TFI^19^. A related receptor belonging to the same sub-family, COUP-TFII was recently shown to be a negative regulator of muscle growth, and could be potential candidates^72, 73^. Future studies using ChIP-sequencing will be needed to delineate both the direct and indirect gene targets of PGC1β in the skeletal muscle. Additional studies with conditional knockout mice will reveal any role PGC1β plays in various muscle wasting conditions including denervation^74^, myotonic dystrophy type 1^75, 76^, and congenital muscular dystrophy^77^, where apoptosis and autophagy have been reported. Finally, the potential muscle deterioration associated with long-term PGC1β activation needs to be considered along with the benefits of PGC1β-activated mitochondrial biogenesis and the oxidative metabolism program.

## Methods

### Mouse husbandry

We have previously described the generation of PGC1β-TG mice (on C57Bl/6 J genetic background) using a muscle-specific human alpha skeletal actin promoter. PGC1β-TG and age-matched WT littermate mice were housed in a temperature controlled room (20–22 °C) with ad libitum access to water and food (Pico Lab rodent diet 20; 13.2% fat) under a 12:12 h light dark cycle. All experiments were performed in male mice. Animals were maintained and treated in accordance with the U.S. National Institute of Health Guide for Care and Use of Laboratory Animals; and the procedures were approved by the Animal Welfare Committee at The University of Texas Medical School in Houston.

### Body mass and body composition

The mice were weighed once a week at the same time of the day. Quantitative nuclear magnetic resonance imaging (EchoMRI 3-in-1 system; Echo Medical System, Houston, TX) was used for measuring fat and lean mass.

### Tissue collection and preparation

Mice were euthanized by isoflurane inhalation followed by cervical dislocation and tissues were rapidly extracted. For RNA, muscles were freeze-clamped in liquid nitrogen. For immunofluorescence, TA or gastrocnemius muscles were mounted in OCT and frozen on melting isopentane in liquid nitrogen.

### Gene expression

Purelink Kit (Ambion, Life technologies, Carlsbad, CA) was used to prepare total mRNA, which was further reverse-transcribed to cDNA with SuperScript III Reverse Transcriptase (Invitrogen, Waltham, MA). Quantitative real-time PCR (qPCR) analysis was performed using SYBR Green PCR Master Mix (Applied Biosystems, Waltham, MA) with an ABI-7900 cycler (Applied Biosystems).

### Western immunoblotting

Muscles were homogenized in Pierce IP Lysis buffer (Thermo Scientific, Waltham, MA) using MagNA Lyser (Roche Applied Sciences, Indianapolis, IN) at speed 5000 followed by centrifugation at 12,000 g for 15 min at 4 °C. The Pierce BCA protein assay kit (Thermo Scientific) was used to quantify protein in supernatant, which was then stored at −80 °C. Samples were separated by SDS-PAGE, transferred onto nitrocellulose membrane, stained using Ponceau S, blocked with 5% milk in phosphate buffered saline with Tween20 (PBST), and incubated overnight at 4 °C with primary antibody (Cell signaling, Danvers, MA – autophagy antibody sampler kit #4445, SQSTM1 #5114, procaspase antibody sampler kit #12742, cleaved PARP #5625, Stat 2 #4597, Stat antibody sampler kit #9939, phospho (p)-Stat antibody sampler kit #9914, pan-AKT #4691, p-AKT ser473 #4060, TSC2 #4308, p-TSC2 Thr1462 #3617, mTOR substrates antibody sampler kit #9862, P70S6K #2708, and β-tubulin #2128, Abcam, Cambridge MA PGC1β #ab176328, Bioss, Woburn, MA - E2F1 #bs-0599R, Ubiquitin #sc8017, Santa Cruz Biotechnology, Dallas, TX). Membranes were then washed with PBST and incubated with the appropriate secondary antibodies (Cell Signaling) for 1 hr. at room temperature, washed with PBST, and bands visualized using chemiluminescence western blotting detection reagents.

For detection of oxidized proteins ab178020 kit from Abcam was used. Samples were homogenized and processed as per manufacturer’s instructions. Briefly, the carbonyl groups in the solubilized protein samples were derivatized using DNPH (2, 4 dinitrophenyl hydrazine) or control solution for 15 min and then neutralized. The samples were then loaded onto SDS PAGE gels and DNP conjugated proteins were detected by western blotting using primary DNP antibody and HRP conjugated secondary antibody. Full-length images are presented in Supplementary Fig. S7.

### Immunohistology

Cryosections (10 μm) were obtained from mid-section of TA muscle. Myosin heavy chains (MHC) type 2a and 2b were stained using the mouse monoclonal antibodies SC71 (5 µg/ml) and BF.F3 (4 µg/ml), respectively (Developmental Studies Hybridoma Bank, Iowa City, IA), as we have previously described^78^. Laminin was stained using antibody from Sigma, St. Louis, MO (L9393) at 1:150 dilution to visualize sarcolemma. Myofiber cross section was calculated using Amira software version 6.3 from FEI. Hillsboro, OR. All primary antibodies were visualized using suitable Alexa Fluor® secondary antibodies from Molecular Probes, Eugene, OR). Frozen WT and PGC1β-TG TA cross sections were analyzed for cleaved caspase 3 using primary antibody from Cell Signaling (9664) at 1:200 dilution. Mounting media containing DAPI (Vectashield H-1500 - Vector Laboratories, Burlingame, CA) was used to visualize nuclei.

### TUNEL assay

Gastrocnemius muscle cryosections (10 μm) were used for the detection of damaged DNA using Dead End Colorimetric TUNEL (Terminal deoxynucleotidyl transferase dUTP Nick End Labeling) assay kit (Promega, Madison, WI- #G7361). The assay was performed following the manufacturer’s instructions with appropriate controls.

### Electron microscopy

Mice were euthanized by isoflurane inhalation followed by cervical dislocation and the TA was isolated, cut into 1mm thick strips and immediately placed in 2% glutaraldehyde (Electron microscopy sciences, Hatfield, PA - #16020) overnight at 4 °C for fixation. Further processing was done at the Cardiovascular Pathology Core Facility (Texas Heart Institute, St. Lukes Episcopal Hospital, Houston, TX, USA). Following fixation, the tissues were washed in 1 M sodium phosphate buffer (pH 7.3), post-fixed in 1% osmium tetroxide for 1 hr and dehydrated through a series of graded alcohol. Tissue samples were then infiltrated with acetone and Polybed 812 plastic resin and embedded in plastic block molds with 100% Polybed 812. Ultra-thin sections (80 nm) were cut from the blocks using a Leica EMUC ultra microtome and mounted on 100 mesh copper grids. Grids were stained with 2% uranyl acetate and Reynold’s lead stain. Grids were placed in JEOL JEM 1250 electron microscope and images were captured on an AMTV600 digital camera. Morphometric quantification of autophagosomes, lysosomes and autophagolysosomes in TA muscles was achieved by visualizing a total of 18 to 21 fields from n = 3 animals per group.

### Indirect calorimetry

Comprehensive Lab Animal Monitoring System (CLAMS; Columbus Instruments, Columbus, OH) was used to measure basal oxygen consumption and CO_2_ liberation in WT and PGC1β-TG mice. Mice were placed in individual cages at room temperature (20 °C–22 °C) with ad libitum access to food and water. They were allowed to acclimate for 72 hours followed by 48 hours of data collection. Food intake and ambulatory activity were also monitored during this period.

### Isolated muscle mitochondrial oxygen consumption

We have previously described the isolation of mitochondria from TA and gastrocnemius as well as the measurement of mitochondrial oxygen consumption using the Seahorse XF^e^96 (Agilent Technologies, Santa Clara, CA)^79^. Respiration was measured using 2 µg mitochondria/well under the following conditions: (i) baseline condition with substrate (10 mM pyruvate and 2 mM malate), (ii) State 3 with substrate and ADP (6 mM) (iii) State 4 with substrate, ADP and oligomycin (2.5 µg/ml), (iv) State 3 u with substrate, ADP, oligomycin and FCCP (12 µM), and (v) Non-mitochondrial respiration with substrate, ADP, oligomycin, FCCP and Antimycin A (4 µM).

### Myofiber isolation and ROS measurements

WT and PGC1β-TG littermates (17–20 weeks old) were anesthetized by isoflurane inhalation and euthanized by cervical dislocation. Subsequent to FDB muscle dissection, myofiber isolation and ROS measurement were performed as previously described with slight modifications^80^. Briefly, FDB muscle was incubated with Dulbecco’s Modified Eagle Medium containing 0.2% Normocin^TM^ (Invitrogen) and 0.4% Collagenase A (Roche Applied Science) for 2 hr at 37 °C. Then FDB muscles were transferred to media containing 10% serum without collagenase and single myofibers were released by triturating using fire polished Pasteur pipettes. Single myofibers were then incubated overnight in 5% CO_2_ at 37 °C. The following day 96 well dishes (Greiner Bio One, Monroe, NC) were coated with extra cellular matrix gel from Engelbreth-HolmSwarm murine sarcoma (Sigma) and plated with FDB myofibers. 4-(2-Hydroxyethyl) piperazine-1-ethanesulfonic acid (HEPES) buffered Ringer’s solution containing (in mM): 146 NaCl, 4.7 KCl, 0.6 MgSO_4_, 1.8 CaCl_2_, 1.6, NaHCO_3_, 0.13 NaH_2_PO_4_, 7.8 glucose, 20 HEPES, pH 7.3 was used to wash the myofibers. Myofibers were then incubated for 30 min with 20 µM 6-carboxy-2, 7-dichlorodihydrofluorescein diacetate (carboxy-H2DCFDA) (Invitrogen) and then de-esterified for 20 min at 37 °C. Fluorescence microscopy was performed with excitation at 488 nm and emission at 560 nm. Baseline unstimulated measurement of 30 sec was followed by electrical stimulation for 5 min (80 Hz, 400 ms pulse train, 0.5 ms pulse width, 1 train every 2 sec) and another 30 sec unstimulated period. Maximal oxidation was obtained by exposing myofibers to 500 µM H_2_O_2_ for 2 min. Myofibers were continuously perfused with buffer during the experiment. Regions of interest (ROI) were drawn on the cell and in the background. The next day a similar protocol was used on remaining myofibers not loaded with carboxy-H2DCFDA to detect mitochondrial ROS production using 5 µM MitoSOX^TM^ (Molecular Probes), except the myofibers were not exposed to H_2_O_2_.

### Data Availibility Statement

The data generated and analyzed during this study is included in this article (and its Supplementary Information files). In addition, we have analyzed previously generated microarray datasets in our laboratory, which are deposited in the NCBI GEO functional genomics public data repository (NCBI GEO #GSE58699).

### Statistics

Unpaired Student’s t test with or without Welch’s correction was used to compare different groups, and p < 0.05 was considered as statistically significant. Data in figures is presented as mean ± SEM.

## Electronic supplementary material

Supplementary Information

## References

[1] Cohen S, Nathan JA, Goldberg AL (2015). Muscle wasting in disease: molecular mechanisms and promising therapies. Nature reviews. Drug discovery.

[2] Agusti AG (2002). Skeletal muscle apoptosis and weight loss in chronic obstructive pulmonary disease. American journal of respiratory and critical care medicine.

[3] Fanzani A, Conraads VM, Penna F, Martinet W (2012). Molecular and cellular mechanisms of skeletal muscle atrophy: an update. Journal of cachexia, sarcopenia and muscle.

[4] Bodine SC (2001). Identification of ubiquitin ligases required for skeletal muscle atrophy. Science.

[5] Nakanishi K, Sudo T, Morishima N (2005). Endoplasmic reticulum stress signaling transmitted by ATF6 mediates apoptosis during muscle development. The Journal of cell biology.

[6] Chabi B (2008). Mitochondrial function and apoptotic susceptibility in aging skeletal muscle. Aging cell.

[7] Bonaldo P, Sandri M (2013). Cellular and molecular mechanisms of muscle atrophy. Disease models & mechanisms.

[8] Baar K (2002). Adaptations of skeletal muscle to exercise: rapid increase in the transcriptional coactivator PGC-1. FASEB journal: official publication of the Federation of American Societies for Experimental Biology.

[9] Russell AP (2003). Endurance training in humans leads to fiber type-specific increases in levels of peroxisome proliferator-activated receptor-gamma coactivator-1 and peroxisome proliferator-activated receptor-alpha in skeletal muscle. Diabetes.

[10] Lin J (2002). Transcriptional co-activator PGC-1 alpha drives the formation of slow-twitch muscle fibres. Nature.

[11] Arany Z (2008). HIF-independent regulation of VEGF and angiogenesis by the transcriptional coactivator PGC-1alpha. Nature.

[12] Handschin C (2007). PGC-1alpha regulates the neuromuscular junction program and ameliorates Duchenne muscular dystrophy. Genes & development.

[13] Mootha VK (2003). PGC-1alpha-responsive genes involved in oxidative phosphorylation are coordinately downregulated in human diabetes. Nature genetics.

[14] Sandri M (2006). PGC-1alpha protects skeletal muscle from atrophy by suppressing FoxO3 action and atrophy-specific gene transcription. Proceedings of the National Academy of Sciences of the United States of America.

[15] Sainz N (2009). Leptin administration favors muscle mass accretion by decreasing FoxO3a and increasing PGC-1alpha in ob/ob mice. PloS one.

[16] Hindi SM (2014). Regulatory circuitry of TWEAK-Fn14 system and PGC-1alpha in skeletal muscle atrophy program. FASEB journal: official publication of the Federation of American Societies for Experimental Biology.

[17] Ruas JL (2012). A PGC-1alpha isoform induced by resistance training regulates skeletal muscle hypertrophy. Cell.

[18] Arany Z (2007). The transcriptional coactivator PGC-1beta drives the formation of oxidative type IIX fibers in skeletal muscle. Cell metabolism.

[19] Yadav V, Matsakas A, Lorca S, Narkar VA (2014). PGC1beta activates an antiangiogenic program to repress neoangiogenesis in muscle ischemia. Cell reports.

[20] Brault JJ, Jespersen JG, Goldberg AL (2010). Peroxisome proliferator-activated receptor gamma coactivator 1alpha or 1beta overexpression inhibits muscle protein degradation, induction of ubiquitin ligases, and disuse atrophy. The Journal of biological chemistry.

[21] Menconi MJ (2010). Sepsis and glucocorticoids downregulate the expression of the nuclear cofactor PGC-1beta in skeletal muscle. American journal of physiology. Endocrinology and metabolism.

[22] Fukawa T (2016). Excessive fatty acid oxidation induces muscle atrophy in cancer cachexia. Nature medicine.

[23] Ginsberg D (2002). E2F1 pathways to apoptosis. FEBS letters.

[24] Polager S, Ofir M, Ginsberg D (2008). E2F1 regulates autophagy and the transcription of autophagy genes. Oncogene.

[25] Wang Q, Shu C, Su J, Li X (2015). A crosstalk triggered by hypoxia and maintained by MCP-1/miR-98/IL-6/p38 regulatory loop between human aortic smooth muscle cells and macrophages leads to aortic smooth muscle cells apoptosis via Stat1 activation. International journal of clinical and experimental pathology.

[26] Carmona-Saez P, Chagoyen M, Tirado F, Carazo JM, Pascual-Montano A (2007). GENECODIS: a web-based tool for finding significant concurrent annotations in gene lists. Genome biology.

[27] Zimmermann KC, Bonzon C, Green DR (2001). The machinery of programmed cell death. Pharmacology & therapeutics.

[28] He C, Klionsky DJ (2009). Regulation mechanisms and signaling pathways of autophagy. Annual review of genetics.

[29] Klionsky DJ (2016). Guidelines for the use and interpretation of assays for monitoring autophagy (3rd edition). Autophagy.

[30] Zhao J, Li X, Guo M, Yu J, Yan C (2016). The common stress responsive transcription factor ATF3 binds genomic sites enriched with p300 and H3K27ac for transcriptional regulation. BMC genomics.

[31] Silva KA (2015). Inhibition of Stat3 activation suppresses caspase-3 and the ubiquitin-proteasome system, leading to preservation of muscle mass in cancer cachexia. The Journal of biological chemistry.

[32] Kang C, Ji LL (2016). PGC-1alpha overexpression via local transfection attenuates mitophagy pathway in muscle disuse atrophy. Free radical biology & medicine.

[33] Cannavino J, Brocca L, Sandri M, Bottinelli R, Pellegrino MA (2014). PGC1-alpha over-expression prevents metabolic alterations and soleus muscle atrophy in hindlimb unloaded mice. The Journal of physiology.

[34] Wenz T, Rossi SG, Rotundo RL, Spiegelman BM, Moraes CT (2009). Increased muscle PGC-1alpha expression protects from sarcopenia and metabolic disease during aging. Proceedings of the National Academy of Sciences of the United States of America.

[35] Vainshtein A, Tryon LD, Pauly M, Hood DA (2015). Role of PGC-1alpha during acute exercise-induced autophagy and mitophagy in skeletal muscle. American journal of physiology. Cell physiology.

[36] Halling, J. F., Ringholm, S., Nielsen, M. M., Overby, P. & Pilegaard, H. PGC-1alpha promotes exercise-induced autophagy in mouse skeletal muscle. *Physiological reports* **4**, doi:10.14814/phy2.12698 (2016). 10.14814/phy2.12698 PMC4758928 26869683

[37] Miura S (2006). Overexpression of peroxisome proliferator-activated receptor gamma co-activator-1alpha leads to muscle atrophy with depletion of ATP. The American journal of pathology.

[38] Cheema N, Herbst A, McKenzie D, Aiken JM (2015). Apoptosis and necrosis mediate skeletal muscle fiber loss in age-induced mitochondrial enzymatic abnormalities. Aging cell.

[39] Park SY (2014). Differential expression of apoptosis-related factors induces the age-related apoptosis of the gracilis muscle in humans. International journal of molecular medicine.

[40] Cea LA (2016). Fast skeletal myofibers of mdx mouse, model of Duchenne muscular dystrophy, express connexin hemichannels that lead to apoptosis. Cellular and molecular life sciences: CMLS.

[41] Yamauchi J, Kumar A, Duarte L, Mehuron T, Girgenrath M (2013). Triggering regeneration and tackling apoptosis: a combinatorial approach to treating congenital muscular dystrophy type 1 A. Human molecular genetics.

[42] Gallot YS (2014). Myostatin gene inactivation prevents skeletal muscle wasting in cancer. Cancer research.

[43] Barreiro E (2011). Inflammatory cells and apoptosis in respiratory and limb muscles of patients with COPD. Journal of applied physiology.

[44] Elmore S (2007). Apoptosis: a review of programmed cell death. Toxicologic pathology.

[45] Argiles JM, Lopez-Soriano FJ, Busquets S (2008). Apoptosis signalling is essential and precedes protein degradation in wasting skeletal muscle during catabolic conditions. The international journal of biochemistry & cell biology.

[46] Micheau O, Solary E, Hammann A, Dimanche-Boitrel MT (1999). Fas ligand-independent, FADD-mediated activation of the Fas death pathway by anticancer drugs. The Journal of biological chemistry.

[47] Gniadecki R (2004). Depletion of membrane cholesterol causes ligand-independent activation of Fas and apoptosis. Biochemical and biophysical research communications.

[48] Garcia-Prat L (2016). Autophagy maintains stemness by preventing senescence. Nature.

[49] Carnio S (2014). Autophagy impairment in muscle induces neuromuscular junction degeneration and precocious aging. Cell reports.

[50] Nascimbeni AC, Fanin M, Masiero E, Angelini C, Sandri M (2012). Impaired autophagy contributes to muscle atrophy in glycogen storage disease type II patients. Autophagy.

[51] He C (2012). Exercise-induced BCL2-regulated autophagy is required for muscle glucose homeostasis. Nature.

[52] Lira VA (2013). Autophagy is required for exercise training-induced skeletal muscle adaptation and improvement of physical performance. FASEB journal: official publication of the Federation of American Societies for Experimental Biology.

[53] Aversa Z (2016). Autophagy is induced in the skeletal muscle of cachectic cancer patients. Scientific reports.

[54] Zhao J (2007). FoxO3 coordinately activates protein degradation by the autophagic/lysosomal and proteasomal pathways in atrophying muscle cells. Cell metabolism.

[55] Mammucari C (2007). FoxO3 controls autophagy in skeletal muscle *in vivo*. Cell metabolism.

[56] Guo Y (2016). AMP-activated kinase alpha2 deficiency protects mice from denervation-induced skeletal muscle atrophy. Archives of biochemistry and biophysics.

[57] Sala D (2014). Autophagy-regulating TP53INP2 mediates muscle wasting and is repressed in diabetes. The Journal of clinical investigation.

[58] Young CN (2015). A novel mechanism of autophagic cell death in dystrophic muscle regulated by P2RX7 receptor large-pore formation and HSP90. Autophagy.

[59] Yamada E (2012). Mouse skeletal muscle fiber-type-specific macroautophagy and muscle wasting are regulated by a Fyn/STAT3/Vps34 signaling pathway. Cell reports.

[60] Doyle A, Zhang G, Abdel Fattah EA, Eissa NT, Li YP (2011). Toll-like receptor 4 mediates lipopolysaccharide-induced muscle catabolism via coordinate activation of ubiquitin-proteasome and autophagy-lysosome pathways. FASEB journal: official publication of the Federation of American Societies for Experimental Biology.

[61] Schiaffino S, Mammucari C (2011). Regulation of skeletal muscle growth by the IGF1-Akt/PKB pathway: insights from genetic models. Skeletal muscle.

[62] Wu Z, Zheng S, Yu Q (2009). The E2F family and the role of E2F1 in apoptosis. The international journal of biochemistry & cell biology.

[63] Ogawa H, Ishiguro K, Gaubatz S, Livingston DM, Nakatani Y (2002). A complex with chromatin modifiers that occupies E2F- and Myc-responsive genes in G0 cells. Science.

[64] Weber JD, Taylor LJ, Roussel MF, Sherr CJ, Bar-Sagi D (1999). Nucleolar Arf sequesters Mdm2 and activates p53. Nature cell biology.

[65] Yan C, Lu D, Hai T, Boyd DD (2005). Activating transcription factor 3, a stress sensor, activates p53 by blocking its ubiquitination. The EMBO journal.

[66] Raffaello A (2010). JunB transcription factor maintains skeletal muscle mass and promotes hypertrophy. The Journal of cell biology.

[67] Bonetto A (2012). JAK/STAT3 pathway inhibition blocks skeletal muscle wasting downstream of IL-6 and in experimental cancer cachexia. American journal of physiology. Endocrinology and metabolism.

[68] Orrenius S, Gogvadze V, Zhivotovsky B (2007). Mitochondrial oxidative stress: implications for cell death. Annual review of pharmacology and toxicology.

[69] Liu Z, Lenardo MJ (2007). Reactive oxygen species regulate autophagy through redox-sensitive proteases. Developmental cell.

[70] Ricci C (2008). Mitochondrial DNA damage triggers mitochondrial-superoxide generation and apoptosis. American journal of physiology. Cell physiology.

[71] Gali Ramamoorthy T (2015). The transcriptional coregulator PGC-1beta controls mitochondrial function and anti-oxidant defence in skeletal muscles. Nature communications.

[72] Lee HJ (2017). Dysregulation of nuclear receptor COUP-TFII impairs skeletal muscle development. Scientific reports.

[73] Xie X, Tsai SY, Tsai MJ (2016). COUP-TFII regulates satellite cell function and muscular dystrophy. The Journal of clinical investigation.

[74] O’Leary MF, Vainshtein A, Carter HN, Zhang Y, Hood DA (2012). Denervation-induced mitochondrial dysfunction and autophagy in skeletal muscle of apoptosis-deficient animals. American journal of physiology. Cell physiology.

[75] Bargiela A (2015). Increased autophagy and apoptosis contribute to muscle atrophy in a myotonic dystrophy type 1 Drosophila model. Disease models & mechanisms.

[76] Loro E (2010). Normal myogenesis and increased apoptosis in myotonic dystrophy type-1 muscle cells. Cell death and differentiation.

[77] Carmignac V (2011). Autophagy is increased in laminin alpha2 chain-deficient muscle and its inhibition improves muscle morphology in a mouse model of MDC1A. Human molecular genetics.

[78] Matsakas A, Yadav V, Lorca S, Evans RM, Narkar VA (2012). Revascularization of ischemic skeletal muscle by estrogen-related receptor-gamma. Circulation research.

[79] Badin PM (2016). Exercise-like effects by Estrogen-related receptor-gamma in muscle do not prevent insulin resistance in db/db mice. Scientific reports.

[80] Pal R (2014). Src-dependent impairment of autophagy by oxidative stress in a mouse model of Duchenne muscular dystrophy. Nature communications.

