# Supplementary material for: Long-term PGC1β overexpression leads to apoptosis, autophagy and muscle wasting
Source: Sci Rep. 2017 Aug 31;7:10237. doi: 10.1038/s41598-017-10238-9 (PMC5578977; doi:10.1038/s41598-017-10238-9)
Supplement: Supplementary file 1 — Supplementary Information [file 41598_2017_10238_MOESM1_ESM.pdf]

Long-term PGC1β overexpression leads to apoptosis, autophagy and muscle wasting

Danesh H. Sopariwala[1], Vikas Yadav[1], Pierre-Marie Badin[1], Neah Likhite[1], Megha Sheth[1,2], Sabina Lorca[1], Isabelle K. Vila[3], Eun Ran Kim[1], Qingchun Tong[1], Min Sup Song[3,4], George G. Rodney[5] and Vihang A. Narkar[1,4,6,*]

[1] Metabolic and Degenerative Diseases, Institute of Molecular Medicine, The University of Texas McGovern Medical School, Houston, TX, 77030, USA
[2] Department of Bioengineering, Rice University, Houston, TX, 77005, USA
[3] Molecular and Cellular Oncology, The University of Texas MD Anderson Cancer Center, Houston, TX, 77030, USA
[4] Graduate School of Biomedical Sciences at The University of Texas Health Science Center at Houston, Houston, TX, 77030, USA
[5] Department of Molecular Physiology and Biophysics, Baylor College of Medicine, Houston, TX, 77030, USA
[6] Integrative Biology and Pharmacology, The University of Texas McGovern Medical School, Houston, TX, 77030, USA

[*]**Corresponding Author:**
Vihang A Narkar, PhD
Metabolic and Degenerative Diseases
Brown Foundation Institute of Molecular Medicine
McGovern Medical School
The University of Texas Health Science Center
1825 Pressler Street
Houston, TX 77030
Phone: +1 713-500-3585
Fax: +1 713-500-2208
Email: vihang.a.narkar@uth.tmc.edu

SUPPLEMENTARY MATERIAL INVENTORY

SUPPLEMENTARY FIGURES

SUPPLEMENTARY FIGURE S1. PGC1β transgene expression.

SUPPLEMENTARY FIGURE S2. Ubiquitin-mediated proteolysis in WT and PGC1β-TG muscles.

SUPPLEMENTARY FIGURE S3. Apoptosis in WT and PGC1β-TG gastrocnemius.

SUPPLEMENTARY FIGURE S4. Autophagy in WT and PGC1β-TG gastrocnemius.

SUPPLEMENTARY FIGURE S5. Basal metabolic rate and skeletal muscle mitochondrial respiration in WT and PGC1β-TG mice.

SUPPLEMENTARY FIGURE S6. AKT/mTOR pathway in WT and PGC1β-TG gastrocnemius.

SUPPLEMENTARY FIGURE S7. Full-length western blots for Figures 4c, 5c and 6b.

SUPPLEMENTARY TABLES

SUPPLEMENTARY TABLE S1. Gene array data showing fold change in up-regulated apoptosis genes in 17 week old PGC1β-TG TA compared to WT TA.

SUPPLEMENTARY TABLE S2. Gene array data showing fold change in up-regulated phagosome genes in 17 week old PGC1β-TG TA compared to WT TA.

SUPPLEMENTARY TABLE S3. Gene array data showing fold change in up-regulated lysosome genes in 17 week old PGC1β-TG TA compared to WT TA.

SUPPLEMENTARY FIGURE S1

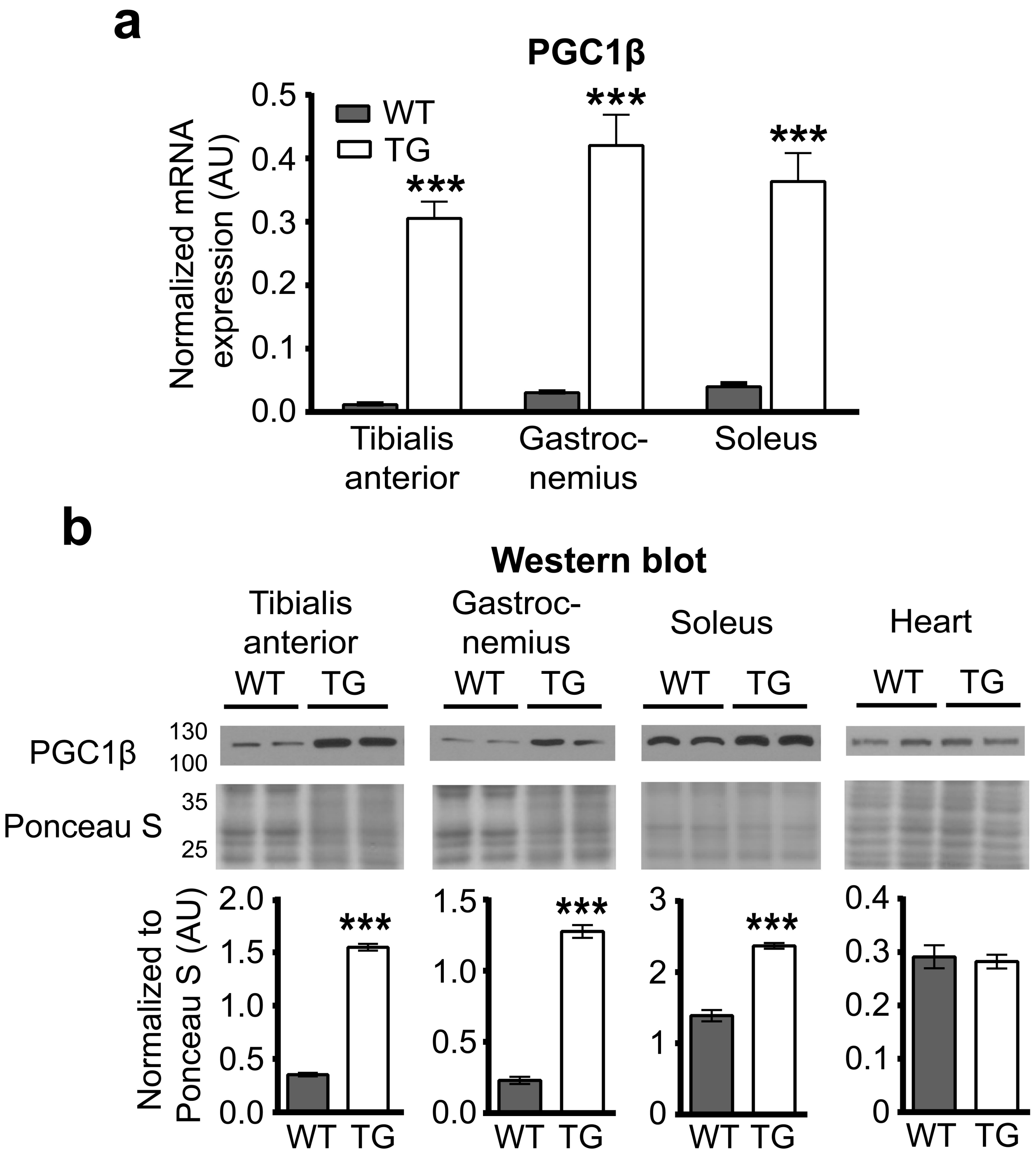

Supplementary Fig. S1. PGC1β transgene expression. (a) mRNA expression of PGC1β in tibialis anterior, gastrocnemius and soleus muscles in 17 week old wild type (WT) and PGC1β-TG (TG) mice (n=5 per group). Expression is normalized to *Eef2* and *Snrpd3*. **(b)** Western blot showing PGC1β protein expression in WT and TG skeletal muscles and heart (n=3 per group). Normalized to Ponceau S. $p < 0.001$ = *** (unpaired Student's t test).

SUPPLEMENTARY FIGURE S2

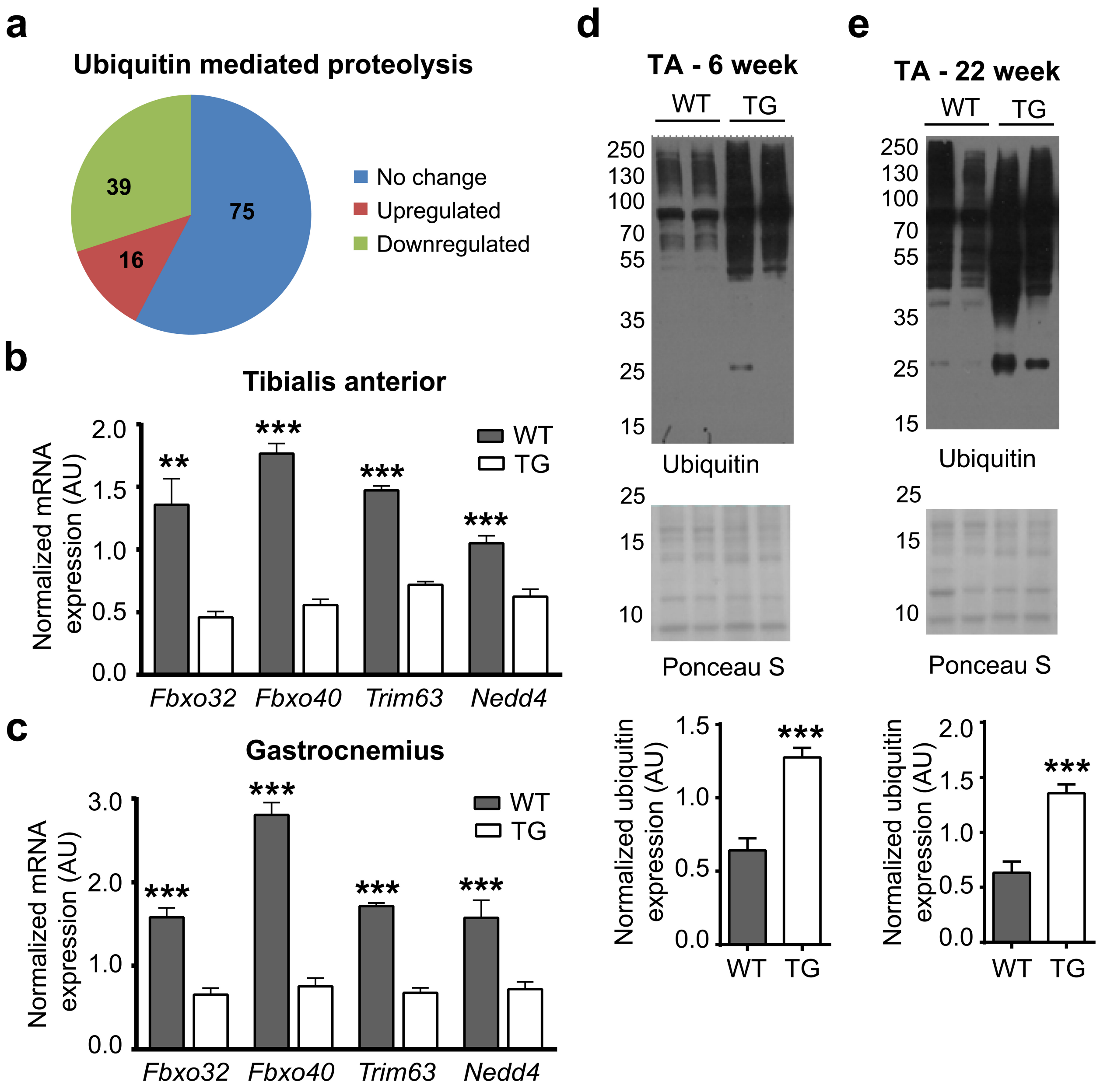

Supplementary Fig. S2. Ubiquitin-mediated proteolysis in WT and PGC1β-TG muscles. (a) KEGG pathway analysis of gene array data from 17 week old wild type (WT) and PGC1β-TG (TG) tibialis anterior (TA) (n=3 mice per group) showing ubiquitin-mediated proteolysis genes. **(b-c)** Quantification of *Fbxo32*, *Fbxo40*, *Trim63* and *Nedd4* mRNA in TA (b) and gastrocnemius (c) from WT and TG mice. (n=5 mice per group). **(d-e)** Western blot showing ubiquitinated protein levels in WT and TG TA from 6 week old (d) and 22 week old mice (e). (n=4 per mice per group). $p<0.01$ = ** and $p<0.001$ = *** (unpaired Student's t test).

SUPPLEMENTARY FIGURE S3

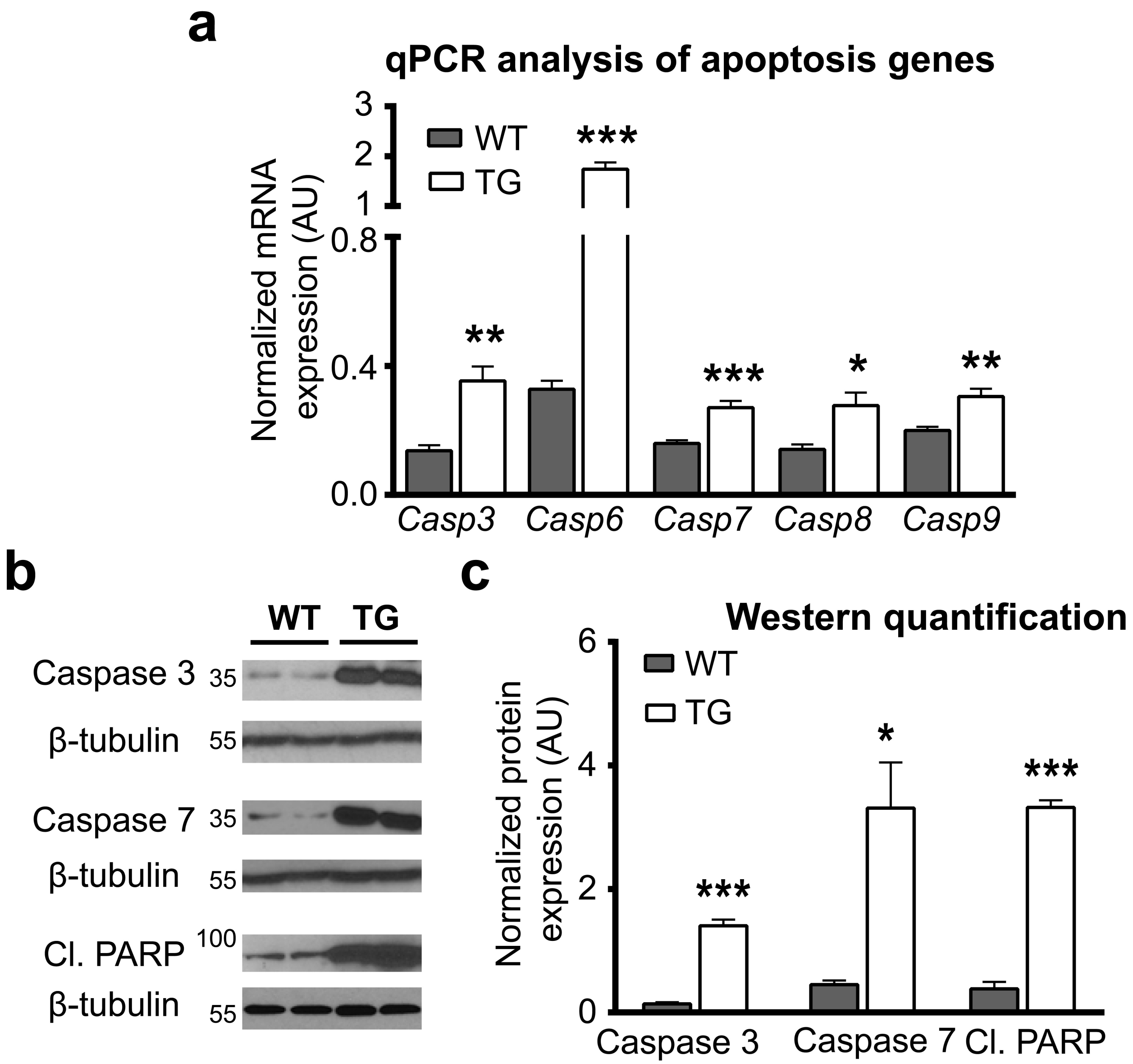

Supplementary Fig. S3. Apoptosis in WT and PGC1β-TG gastrocnemius. (a) mRNA expression of various caspases (Casp) in gastrocnemius of 17 week old wild (WT) and PGC1β-TG (TG) mice (n=5 per group). mRNA expression is normalized to *Eef2* and *Snrpd3*. **(b)** Representative western blots showing protein expression of Caspase 3 & 7 and cleaved poly ADP ribose polymerase (Cl. PARP) in 17 week old WT and TG gastrocnemius muscles. **(c)** Quantification of western blots from (b) (n=3 mice per group). Normalized to β-tubulin. $p<0.05$ = *, $p<0.01$ = ** and $p<0.001$ = *** (unpaired Student's t test).

SUPPLEMENTARY FIGURE S4

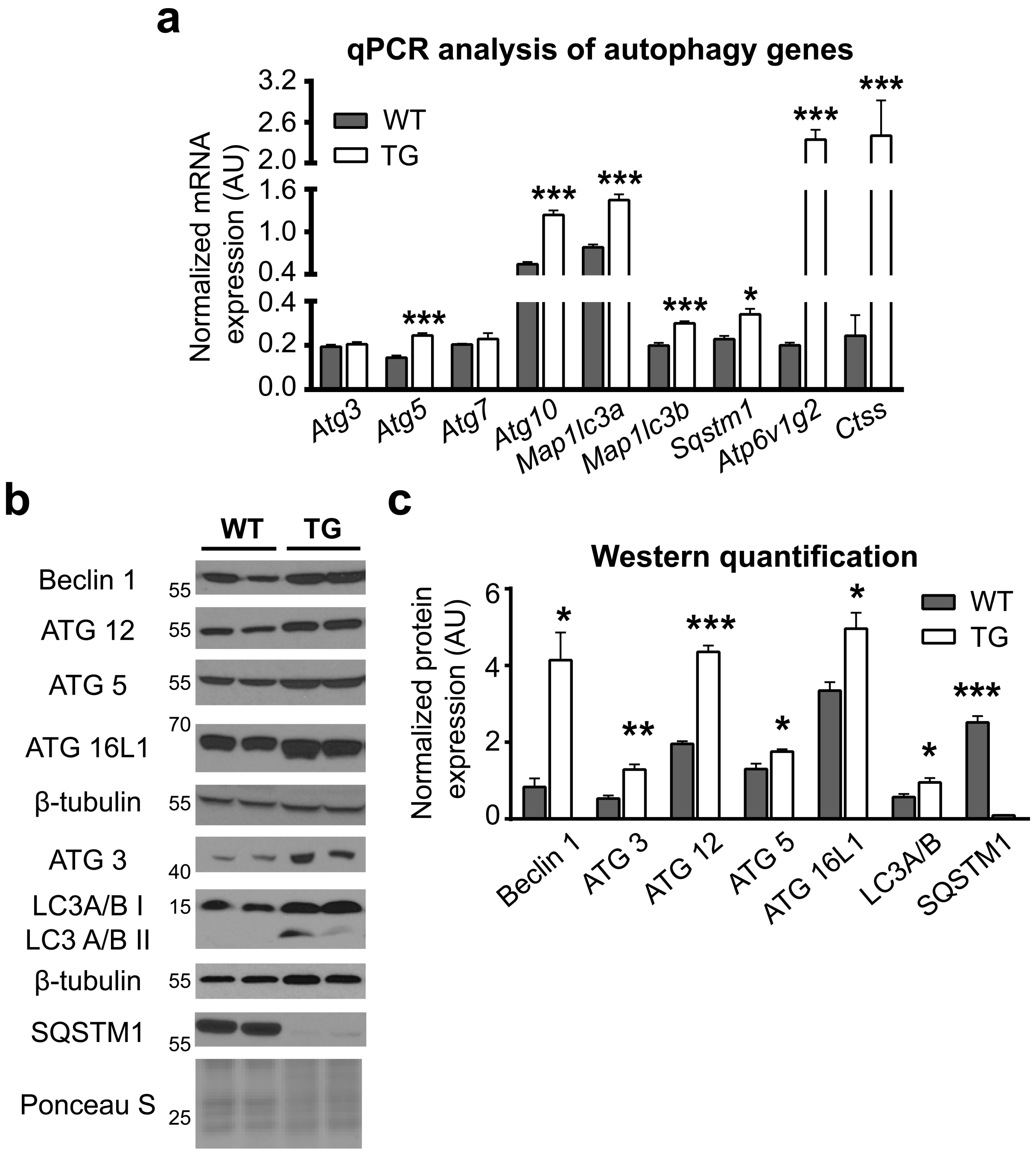

Supplementary Fig. S4. Autophagy in WT and PGC1β-TG gastrocnemius. (a) mRNA expression of autophagy and lysosomal genes in gastrocnemius of 17 week old wild type (WT) and PGC1β-TG (TG) mice (n=5 per group). **(b)** Representative western blots showing protein expression of Beclin1, Autophagy related (ATG) 3, 5, 12, 16L1, LC3A/B I and II, and Sequestosome 1 (SQSTM1) in 17 week old WT and TG gastrocnemius. **(c)** Quantification of protein expression from (b). n=3 per group. p<0.05 = *, p<0.01 = ** and p<0.001 = *** (unpaired Student's t test).

SUPPLEMENTARY FIGURE S5

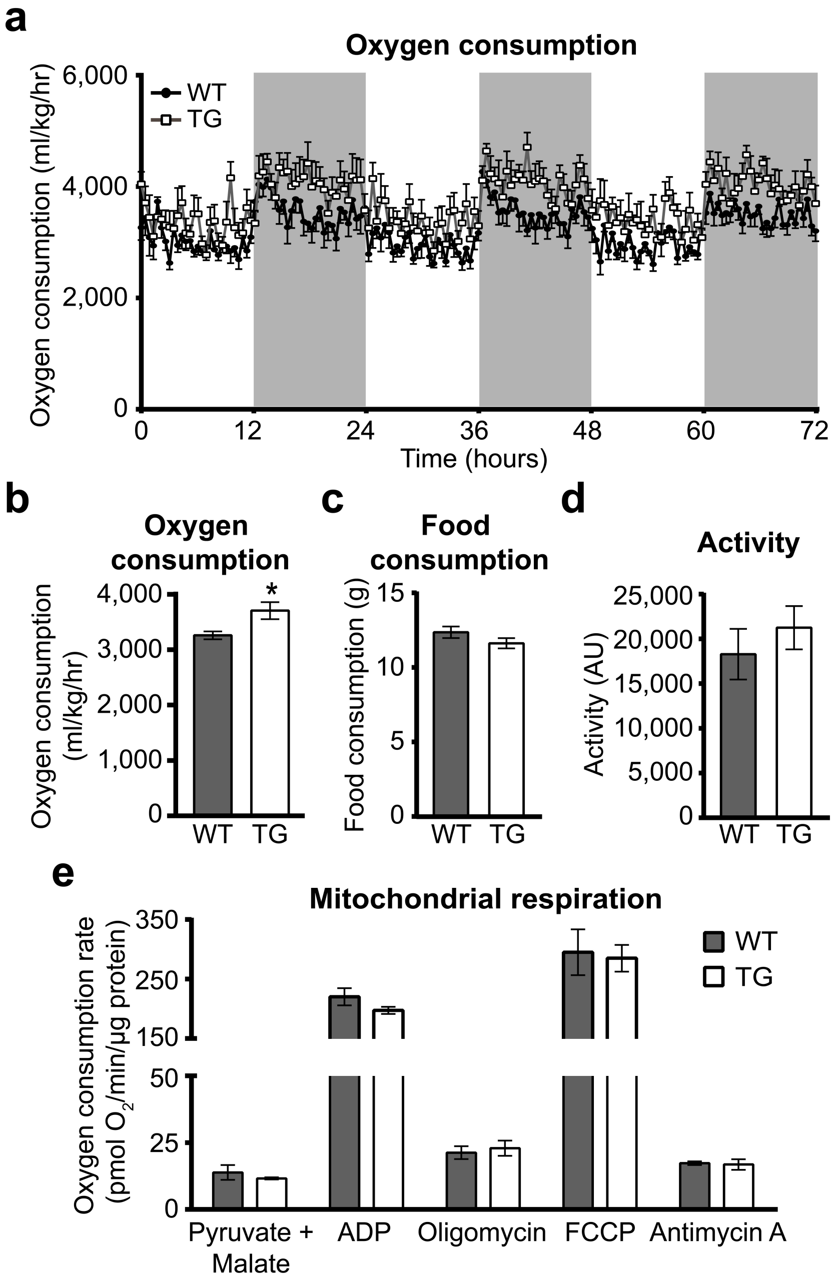

Supplementary Fig. S5. Basal metabolic rate and skeletal muscle mitochondrial respiration in WT and PGC1β-TG mice. (a) Graph showing comparison of oxygen consumption pattern during light and dark (grey shadow) period for 17 week old wild type (WT) and PGC1β-TG (TG) mice (n=6 per group). **(b)** Oxygen consumption in WT and TG mice. **(c-d)** Food consumption (c) and ambulatory activity (d) in WT and TG mice. **(e)** Quantification of the different states of respiration in presence of pyruvate and malate as substrate in response to ADP (State 3), Oligomycin (State 4_0), FCCP (State 3_u) and Antimycin A (State 5) showing that there is no difference in the rate of oxygen consumption during these different states between 17 to 20 week old wild type (WT) and PGC1β-TG (TG) mitochondria isolated from the fast twitch hind limb skeletal muscles (n=3 mice per group, triplicate experiments for each mouse).AU – arbitrary units. $p < 0.05$ = * (unpaired Student's t test).

SUPPLEMENTARY FIGURE S6

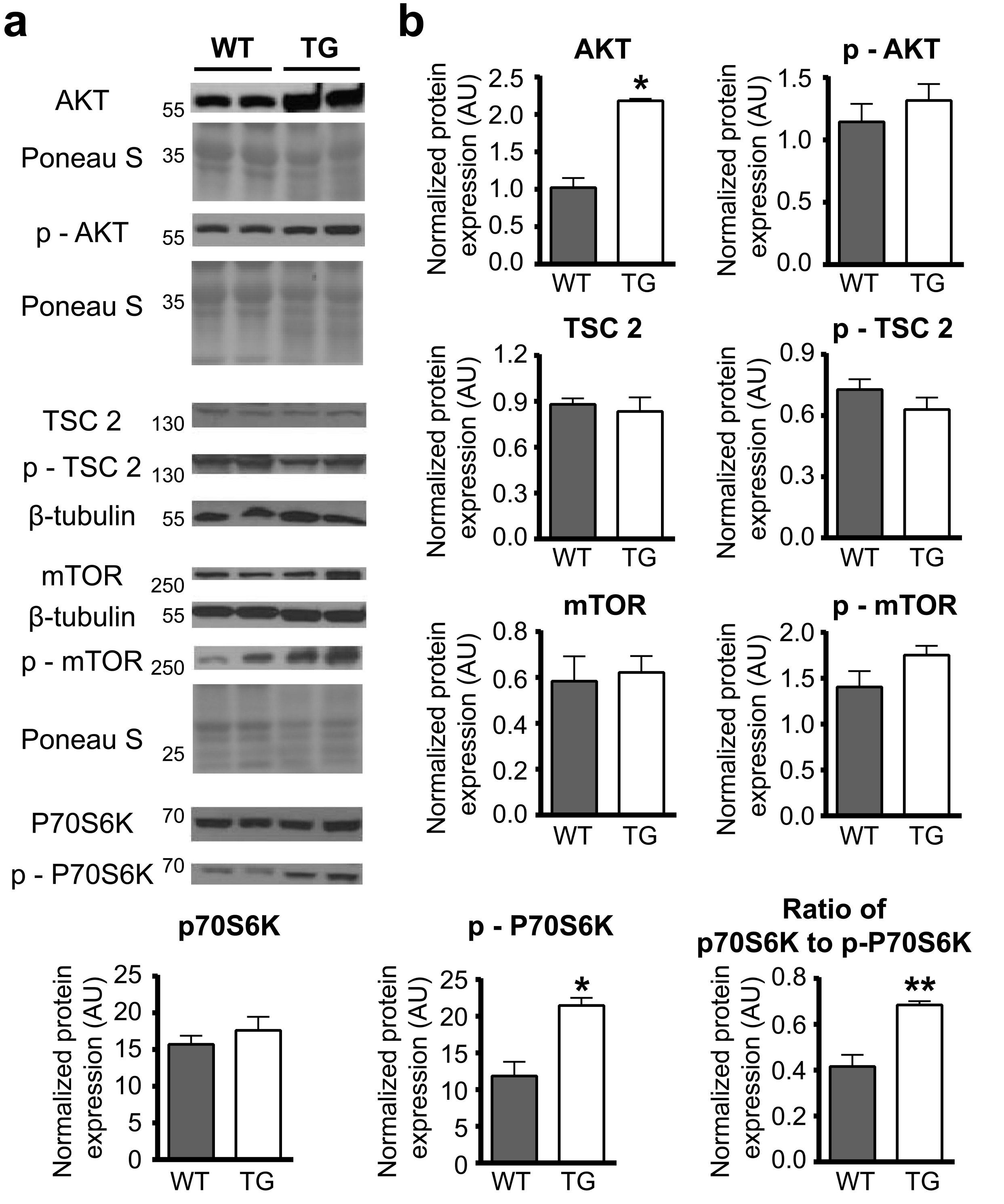

Supplementary Fig. S6. AKT/mTOR pathway in WT and PGC1β-TG gastrocnemius. (a) Representative western blots for Akt/mTOR pathway proteins from 17 week old wild type (WT) and PGC1β-TG (TG) gastrocnemius muscle showing total and phosphorylated protein levels of AKT, TSC2 (tuberous sclerosis 2), mTOR (mammalian target of Rapamycin) and P70S6K. **(b)** Quantification of the western blots from (a). (n=3 per group). Protein expression normalized to Ponceau S or β-tubulin. $p<0.05$ = *, and $p<0.01$ = ** (unpaired Student's t test).

SUPPLEMENTARY FIGURE S7

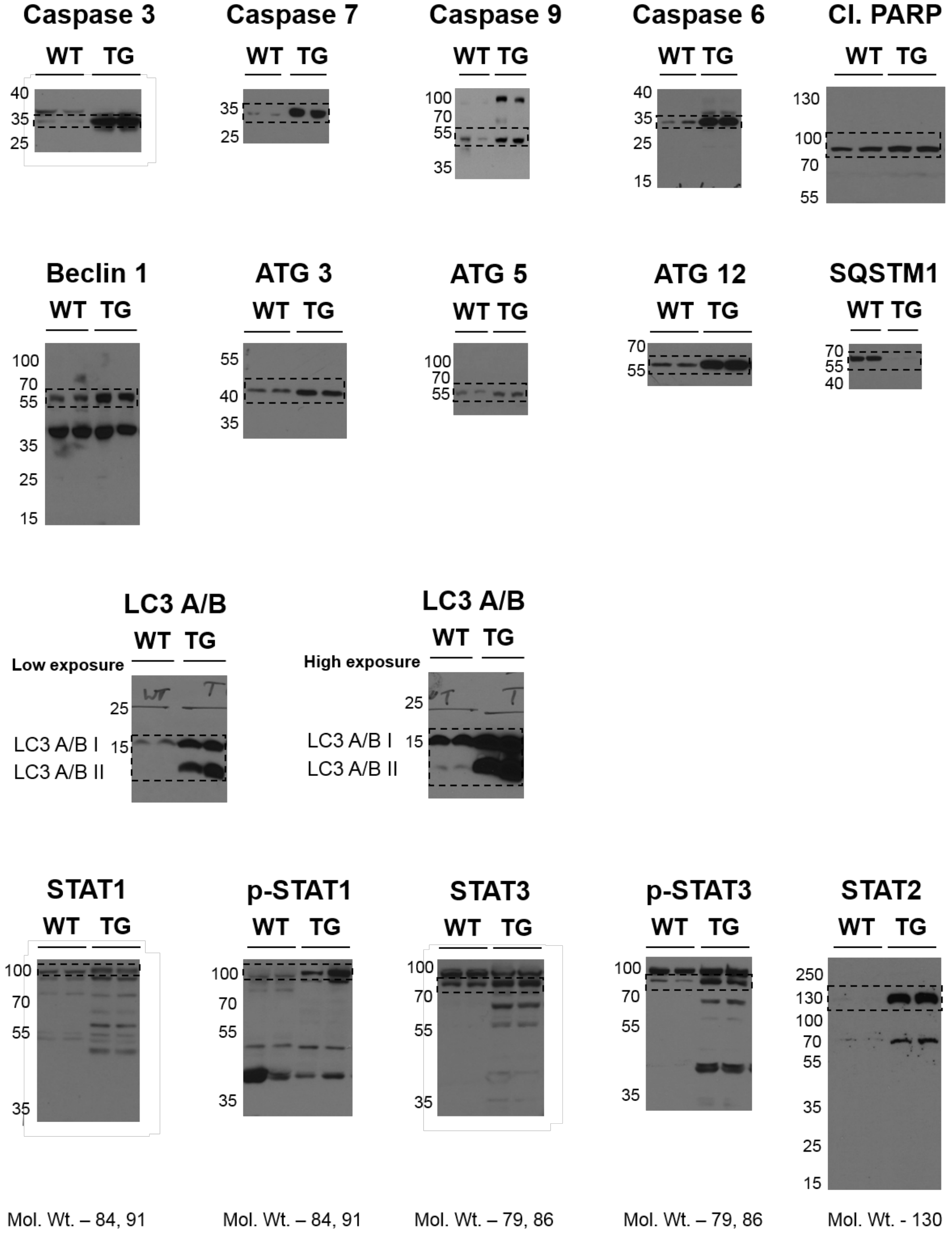

Supplementary Fig. S7. Full length western blots. Full-length western blots for figure 4c, figure 5c and figure 6b. The dotted box region is the cropped portion of the blot shown in the main figures.

SUPPLEMENTARY TABLE S1. Gene array data showing fold change in up-regulated apoptosis genes in 17 week old PGC1β-TG TA compared to WT TA (n=3 per group).

Gene Symbol	Fold Change	Gene Name
Bid	10.556	BH3 interacting domain death agonist
Prkar2b	3.95	protein kinase, cAMP dependent regulatory, type II beta
Pik3cg	3.803	phosphoinositide-3-kinase, catalytic, gamma polypeptide
Casp6	3.377	caspase 6
Il3ra	2.964	interleukin 3 receptor, alpha chain
Irak2	2.472	interleukin-1 receptor-associated kinase 2
Myd88	2.443	myeloid differentiation primary response gene 88
Casp8	2.357	caspase 8
Tnrsf1a	2.269	tumor necrosis factor receptor superfamily, member 1a
Ppp3cc	1.872	protein phosphatase 3, catalytic subunit, gamma isoform
Bad	1.773	BCL2-associated agonist of cell death
Fadd	1.766	Fas (TNFRSF6)-associated via death domain
Apaf1	1.74	apoptotic peptidase activating factor 1
Endod1	1.637	endonuclease domain containing 1
Prkar1a	1.577	protein kinase, cAMP dependent regulatory, type I, alpha
Aifm1	1.562	apoptosis-inducing factor, mitochondrion-associated 1
Trp53	1.558	transformation related protein 53
Nfkb1	1.514	nuclear factor of kappa light polypeptide gene enhancer in B cells 1, p105
Map3k14	1.479	mitogen-activated protein kinase kinase kinase 14
Prkacb	1.389	protein kinase, cAMP dependent, catalytic, beta
Capn2	1.357	calpain 2
Casp9	1.334	caspase 9
Pik3r1	1.309	phosphatidylinositol 3-kinase, regulatory subunit, polypeptide 1(p85 alpha)
Ikbkg	1.261	inhibitor of kappaB kinase gamma

SUPPLEMENTARY TABLE S2. Gene array data showing fold change in up-regulated phagosome genes in 17 week old PGC1β-TG TA compared to WT TA (n=3 per group).

Gene Symbol	Fold Change	Gene Name
Cd68	12.909	CD68 molecule
Ctss	10.383	cathepsin S
Tcirg1	5.471	T-cell, immune regulator 1, ATPase, H+ transporting, lysosomal V0
Igmn	4.833	legumain
Ctsh	4.225	cathepsin H
Napsa	4.173	napsin A aspartic peptidase
Slc11a1	3.977	solute carrier family 11 (proton-coupled divalent metal ion
Hyal1	3.452	hyaluronoglucosaminidase 1
Ctsk	3.261	cathepsin K
Ctsa	2.606	cathepsin A
Arsb	2.404	arylsulfatase B
Ppt1	2.209	palmitoyl-protein thioesterase 1
Hexb	2.206	hexosaminidase B (beta polypeptide)
Gusb	2.19	glucuronidase, beta
Abca2	2.026	ATP-binding cassette, sub-family A (ABC1), member 2
Ctsc	1.98	cathepsin C
Gm2a	1.979	GM2 ganglioside activator
Ctsf	1.978	cathepsin F
Abcb9	1.849	ATP-binding cassette, sub-family B (MDR/TAP), member 9
Ctsb	1.828	cathepsin B
Asah1	1.798	N-acylsphingosine amidohydrolase (acid ceramidase) 1
Manba	1.616	mannosidase, beta A, lysosomal
Psap	1.603	prosaposin
Acp2	1.574	acid phosphatase 2, lysosomal
Ap1m1	1.488	adaptor-related protein complex 1, mu 1 subunit
Gga3	1.409	golgi-associated, gamma adaptin ear containing, ARF binding protein
M6pr	1.399	mannose-6-phosphate receptor (cation dependent)
Clta	1.384	clathrin, light chain A
Atp6v0a2	1.353	ATPase, H+ transporting, lysosomal V0 subunit a2
Ap4b1	1.321	adaptor-related protein complex 4, beta 1 subunit
Igf2r	1.185	insulin-like growth factor 2 receptor

SUPPLEMENTARY TABLE S3. Gene array data showing fold change in up-regulated lysosome genes in 17 week old PGC1β-TG TA compared to WT TA (n=3 per group).

Gene Symbol	Fold Change	Gene Name
Fcgr4	52.462	Fc receptor, IgG, low affinity IV (CD16-2)
Fcgr1	22.035	Fc receptor, IgG, high affinity I (CD64)
Clec7a	15.044	C-type lectin domain family 7, member A
Thbs3	13.211	thrombospondin 3
Atp6v1g2	9.79	ATPase, H+ transporting, lysosomal 13kDa, V1 subunit G2
Ncf4	8.789	neutrophil cytosolic factor 4, 40kDa
Fcgr2b	7.717	Fc fragment of IgG, low affinity IIb, receptor (CD32)
H2-t10	7.545	histocompatibility 2, T region locus 10
H2-ab1	7.471	histocompatibility 2, class II antigen A, beta 1
Coro1a	7.408	coronin, actin binding protein, 1A
H2-dmb2	7.397	histocompatibility 2, class II, locus Mb2
H2-eb1	6.089	histocompatibility 2, class II antigen E beta
Tlr2	5.596	toll-like receptor 2
Tcirg1	5.471	T-cell, immune regulator 1, ATPase, H+ transporting, lysosomal V0 subunit A3
H2-dmb1	5.113	histocompatibility 2, class II, locus Mb1
H2-aa	4.757	histocompatibility 2, class II antigen A, alpha
Fcgr3	4.611	Fc receptor, IgG, low affinity III
H2-t23	4.499	histocompatibility 2, T region locus 23
H2-q8	4.38	histocompatibility 2, Q region locus 8
Tlr4	4.237	toll-like receptor 4
Mrc1	4.231	mannose receptor, C type 1
H2-q6	4.133	histocompatibility 2, Q region locus 6
H2-dma	4.132	histocompatibility 2, class II, locus DMa
Tap1	4.083	transporter 1, ATP-binding cassette, sub-family B (MDR/TAP)
H2-q7	4.05	histocompatibility 2, Q region locus 7
Cd14	3.888	CD14 molecule
H2-k1	3.782	histocompatibility 2, K1, K region
Tubb6	3.779	tubulin, beta 6
Cyba	3.645	cytochrome b-245, alpha polypeptide
H2-d1	3.417	histocompatibility 2, D region locus 1
5430435g22rik	3.392	RIKEN cDNA 5430435G22 gene
Comp	3.247	cartilage oligomeric matrix protein
Atp6v0e	2.764	ATPase, H+ transporting, lysosomal V0 subunit E
Itga5	2.71	integrin, alpha 5 (fibronectin receptor, alpha polypeptide)
Atp6v0e	2.764	ATPase, H+ transporting, lysosomal V0 subunit E

Gene Symbol	Fold change	Gene Name
Itga5	2.71	integrin, alpha 5 (fibronectin receptor, alpha polypeptide)
Tubb2b	2.657	tubulin, beta 2B
Tuba1b	2.628	tubulin, alpha 1b
H2-m3	2.499	histocompatibility 2, M region locus 3
C3	2.191	complement component 3
Atp6v1d	1.91	ATPase, H+ transporting, lysosomal 34kDa, V1 subunit D
Tubb5	1.851	tubulin, beta 5
Tap2	1.735	transporter 2, ATP-binding cassette, sub-family B (MDR/TAP)
Tfrc	1.706	transferrin receptor (p90, CD71)
Sec61a1	1.639	Sec61 alpha 1 subunit (S. cerevisiae)
Lamp2	1.583	lysosomal-associated membrane protein 2
Tuba1a	1.527	tubulin, alpha 1a
Dync1li2	1.52	dynein, cytoplasmic 1, light intermediate chain 2
Rab5c	1.509	RAB5C, member RAS oncogene family
Atp6v0a2	1.353	ATPase, H+ transporting, lysosomal V0 subunit a2
Atp6v1h	1.286	ATPase, H+ transporting, lysosomal 50/57kDa, V1 subunit H
Stx7	1.251	syntaxin 7
Sec22b	1.152	SEC22 vesicle trafficking protein homolog B (S. cerevisiae) (gene/pseudogene)
